# Application of Nanotechnology in Wood-Based Products Industry: A Review

**DOI:** 10.1186/s11671-020-03438-2

**Published:** 2020-11-04

**Authors:** Latifah Jasmani, Rafeadah Rusli, Tumirah Khadiran, Rafidah Jalil, Sharmiza Adnan

**Affiliations:** Forest Products Division, Forest Research Institute Malaysia (FRIM), 52109 Kepong, Selangor Malaysia

**Keywords:** Forest product, Wood, Nanotechnology, Nanocellulose, Nanomaterial

## Abstract

Wood-based industry is one of the main drivers of economic growth in Malaysia. Forest being the source of various lignocellulosic materials has many untapped potentials that could be exploited to produce sustainable and biodegradable nanosized material that possesses very interesting features for use in wood-based industry itself or across many different application fields. Wood-based products sector could also utilise various readily available nanomaterials to enhance the performance of existing products or to create new value added products from the forest. This review highlights recent developments in nanotechnology application in the wood-based products industry.

## Introduction

Nanotechnology is defined as the manipulation of matters between 1 and 100 nm. Nanotechnology is a multidisciplinary field that combines science and technology with aims to develop new and improved materials with significant functions, physical and chemical properties [1, 2]. Material at this scale has unique properties compared to the same matters at higher dimensions [3]. For this reason, various innovative applications through nanotechnology can be explored in many disciplines.

Not only as a technology enabler, nanotechnology can also be a driver towards achieving national economic growth. The forest products industry in Malaysia contributes around RM22.5 billion value of export in 2019 [4]. Wood products exported from Malaysia include sawn timbers, veneers, plywoods and mouldings. The wood-based industry in Malaysia has the opportunity to fully exploit the nanotechnology to its own advantages. It is imperative that the effort to apply nanotechnology in the wood-based industry particularly in Malaysia is supported and given attention in order to diversify and add value to the existing timber products which eventually will boost the economic growth in this arena.

The fact that forest plays an essential role for civilisation for so many years cannot be ruled out as it has become the source of lignocellulosic materials for various product exploitations and development. As a matter of fact, wood products have long been available in the society in the form of timbers, furniture, papers and many other functional materials [5, 6]. The application of nanotechnology in forestry sector particularly in forest products or wood-based products warrants an attention from all related players and stakeholders. Nanotechnology utilisation could result in stronger, multifunctional yet lighter wood-based products [3]. Traditional forest products such as pulp and paper, wood composites, wood coatings and wood preservatives can be expanded or transformed into new or valued added products to find broader and/or more advanced applications [5].

In this review paper, the application of nanotechnology in wood-based products industry can be divided into two [2] pathways:

1. Derivation of nanomaterial(s) from the forest
  Increasing concerns with respect to the environment have triggered an immense need to introduce sustainable and biodegradable nanosized material. This new material called nanocellulose can be produced from forest resources in a safe and sustainable manner. The abundance of lignocellulosic material from the forest has created a surge of interest to convert cellulose to nanocellulose. Nanocellulose is principally cellulose at molecular level that has several key features such as high strength and stiffness, high strength-to-weight ratio, electromagnetic response and a large surface area [5, 7–9]. The outstanding characteristics of this material offer huge application potentials beyond forest products industry itself. In wood-based product area, it can be applied as a reinforcing agent in pulp and paper and wood composite or as a coating material in wood coating [10–12]. Due to its versatility, nanocellulose could be used as one of the components (as substrate, stabiliser, electrode) for non-forest products sector such as electronics, sensors, batteries, food, pharmaceutical and cosmetics [5, 13].
2. Use of nanomaterial(s) for wood-based products
  Nanomaterials could be used to enhance existing wood-based products in terms of its functionality. For example, the use of nanomaterial in wood coating such as nanozinc oxide or nanotitanium oxide can enhance the functionality of the wood in terms of its durability, fire resistance and UV absorption as well as decrease of water absorption [14–16]. On the other hand, the application of nanoencapsulation in wood preservative [17, 18] could improve the impregnation of wood with pesticides by ensuring that chemicals can penetrate deeper into the wood, thus reducing the issue of excessive leaching [19]. This improves the durability of treated woods against biodegradation agents.

A number of review articles on the application of nanotechnology in forestry and forest products have been published [20, 21]. An article by McCrank [21] gives an overall overview of nanotechnology application in forest sector, whereas Moon et al. [20] discuss more on nanoindentation. To the best of the authors’ knowledge, there are limited publications on this topic particularly covering selected sectors of wood-based industry. This review paper will provide a highlight on the recent progress of nanotechnology applications on the selected wood-based sectors, namely pulp and paper, wood composite, wood coating and wood durability. Moreover, some potential applications of nanocellulose being a new generation of cellulose in wood-based products industry are also highlighted in areas such as energy and sensor.

## Applications

### Pulp and Paper

The annual production of paper and paperboard worldwide is more than 400 million tonnes [22]. It shows that the current digital era has not stopped the continual use of paper products in the community. Nevertheless, the rising demand is mostly for various packaging products [23]. This rise may be spurred by the shift of consumer preference over online shopping and the boost of e-commerce that gives such convenience to shop at the comfort of one’s home and has the product delivered safely to the intended recipients.

Paper is made from lignocellulosic materials that have undergone a series of primary processing, treatment, papermaking, drying and coating. The papermaking stage is where all the mixing of pulp and additives is added and adjusted to suit different end products. Nanotechnology is applied in pulp and paper in the form of nanomaterials or nanoadditives. For example, forest resources or rather lignocellulosic materials can be exploited to extract the nanosized building block or known as nanocellulose that serves as a reinforcement unit to provide unique mechanical strength, functionality and flexibility [5]. This new generation of cellulose is produced via cell wall delamination to obtain nanofibril or by extraction of crystalline cellulose at the nanoscale. Nanocellulose is added into papermaking mainly to increase the paper strength. Other nanomaterials used during paper production process include nanosilica, nanozeolite and etc. [22]. The incorporation of nanomaterials or nanoadditives in paper industry can help to improve the performance of the paper products.

Main additives used or can potentially be used in the pulp and paper industry are discussed in the following sections.

#### Nanocellulose as Wet or Dry Strength Agent

**Fig. 1:**
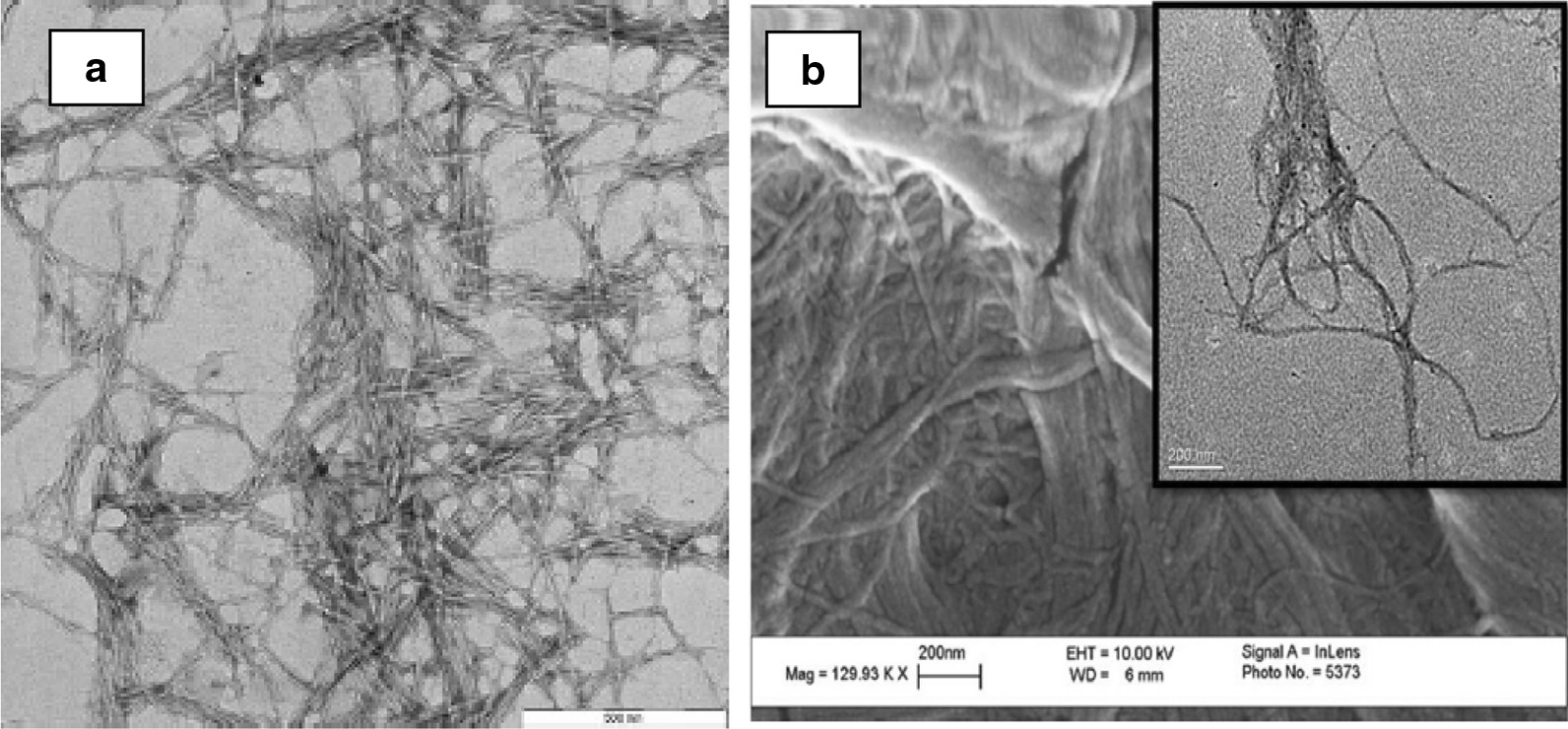
TEM image of NCC from *Acacia magium* **a** *Source*: Jasmani and Adnan [26]. Reproduced with permission of Elsevier and NFC from softwood pulp, **b** *Source*: Zhao et al. [27]. Reproduced with permission of Elsevier

Nanocellulose has been extensively studied as a strength additive in pulp and paper field [24, 25]. Nanocellulose is getting tremendous interests due to its abundance, availability and also because of its interesting characteristics such as biodegradable, low toxicity, excellent mechanical and optical properties, high surface area and renewability [5, 7]. Nanocellulose is actually cellulose at a nanoscale dimension which can be isolated and prepared from any lignocellulosic materials that includes wood pulp, non-wood plants, wood and agricultural residues, bacterial cellulose and tunicates. Cellulose is a linear compound with β-1,4 linked glucose units that comprises crystalline and amorphous regions. The removal of amorphous domains from cellulose chain using mineral acid leads to the isolation of nanocrystalline cellulose, whereas cell wall delamination via high shear mechanical action results in the reduction of cellulose width thus forming nanofibrillated cellulose. These two categories of nanocellulose are the most studied nanomaterial in pulp and paper field for product enhancement. For the last decade, numerous works have been focused on the preparation of nanofibrillated cellulose (NFC) and nanocrystalline cellulose (NCC) from various plant resources. Figure 1 shows images of nanocrystalline cellulose and nanofibrillated cellulose using transmission electron microscopy (TEM).

The properties of nanocellulose vary according to preparation methods and source of cellulose. Table 1 shows the morphology and crystallinity of nanofibrillated cellulose and nanocrystalline cellulose.

**Table 1:** Dimension of nanocellulose from different sources

Source	Types of nanocellulose	Length	Width (nm)	References
Hardwood pulp	Nanofibrillated cellulose	Not determined	5–35	[28]
	Nanocrystalline cellulose	100–150 nm	4–5	[29]
Softwood pulp	Nanofibrillated cellulose	Not determined	16–28	[27]
	Nanocrystalline cellulose	100–200 nm	4–5	[29]
Agricultural crop	Nanofibrillated cellulose	Not determined	10–90	[30]
	Nanocrystalline cellulose	100–500 nm	3–5	[31]

##### From Cellulose to Nanocellulose

Nanofibrillated cellulose is the most studied nanoadditive in papermaking as many works have been reported [3, 25]. Nanofibrillated cellulose is prepared by subjecting fibres to selective high shear mechanical treatments. There are many methods and combination of methods to produce nanofibrillated cellulose. Homogenisation and microfluidisation are the common techniques used for nanofibrillated cellulose preparation which involves subjecting the fibres into a small nozzle under high pressure for repeated passes usually between 10 and 15 passes [32, 33]. This type of treatment if used alone requires high energy consumption, thus pre-treatment with chemicals such as TEMPO (2,2,6,6-tetramethylpiperidin-1-oxyl)-mediated oxidation [34], carboxymethylation or enzyme [35, 36] is usually required to save energy. Using this method, the nanofibrillated cellulose is produced as a result of high shear rates induced by high velocity and force from the fluid stream. Another popular method to produce nanofibrillated cellulose is via the grinding method. The grinding machine consists of static and rotating stone disks. The shearing force generated by the stones breaks down the cell wall fibres to individualised nanofibrils. To avoid a wide distribution in width using this method due to aggregation [37], the authors recommended that the feedstocks need to be kept in water after pulping and bleaching processes. This is to hinder the formation of hydrogen bond network when it is at a dry state [37, 38]. The ultrasonication method has also been studied to prepare nanofibres. Sonication involves the generation of cavitational high shear force from ultrasonic waves which can break down the fibre cell structure [39]. The sonication technique is said to produce well-dispersed and stable nanofibres [3]. Besides that, cryo-crushing can also be applied to convert native fibres to nanofibres. The fibres need to be frozen using liquid nitrogen followed by high shear forces. The high shear force induced pressure on the ice crystals that force cell walls to rupture and release the microfibril [40–43].

Nanocrystalline cellulose extracted from native cellulose is commonly prepared using acid hydrolysis. Other methods involving the use of specific chemical such as TEMPO-mediated oxidation [44] and ionic liquids [45] have also been studied for nanocellulose preparation. An application of cellulase enzyme has also been reported [46, 47].

During acid hydrolysis, the degree of polymerisation of cellulose decreases rapidly, but it stabilises at some point known as the level off degree of polymerisation [48]. The reason for this behaviour could be due to amorphous regions being rapidly hydrolysed by the acid. As hydrolysis starts, acid preferably attacks the amorphous region due to its high volume and hydrolyses the easily accessible glycosidic bond [49]. Once it hydrolyses the easily accessible bond, further hydrolysis occurs at a much slower rate at the reducing end of the glucose chain and on crystalline region surface [50]. Acid hydrolysis is influenced by factors such as acid type, acid concentration, temperature and time [49, 51, 52]. Any change in acid concentration, hydrolysis time and temperature condition has an effect on the morphology of the nanocrystalline cellulose. For example, increasing both hydrolysis time [53] and acid concentration [29] leads to shorter nanocrystalline cellulose, whereas high temperature results in complete conversion of cellulose to glucose [50, 54, 55].

The addition of nanocellulose in papermaking leads to a paper of better performance as to which it increases paper strength [56, 57] and density [58–60] and also reduces porosity [61]. The addition of nanostructured cellulose results in similar paper properties added with beaten fibres [3, 25]. In theory, the strength of paper can be increased by the inclusion of wet and dry strength agents [24, 62], addition of functionalised fibre [63] and beating [64]. The enhancement results in fibre bonding ability [25]. Boufi et al. [25] proposed the mechanism may be due to the increase in bonded area as a result of nanocellulose acting as a connector between fibres that leads to fibre-to-fibre bonding. Besides that, it could be also due to different networks created in the fibre that results in the increase in bonding capability. Given the micrometer length of nanocellulose they can act as a bridge to connect the neighbouring fibres that leads to stronger network [25]. The mutual bonding network attributed by fibre and nanocellulose increases the strength of the paper.

Nanocellulose as nanoadditive improves internal bonding that results in an increase in dry tensile strength, reduction in air permeability and opacity, and higher density [3]. Being a nanoscale material, nanocellulose has a high surface area, and thus hydrogen bonding can be formed more effectively. It is added as a wet end additive for dry strength enhancement and retention improvement. Nanocellulose commonly used for dry strength additive is in the form of nanofibrillated cellulose.

There are different strategies to add NFC into the pulp furnish as it can be added directly with [24, 62, 65] or without retention aid [58] or mixed with other fillers or long fibres [66, 67] followed with a retention aid. An increase in tensile strength after NFC addition relates to the amount added. It was found that an increase of 5% tensile strength was observed when 3% NFC was added into the pulp furnish composed of beaten pulp together with cationic starch [24]. It is interesting to note that the effect was more remarkable if the pulp is less beaten. For instance, more than 100% increase in tensile strength when 6% NFC was added to thermomechanical pulp. On the other hand, NFC had a lesser impact when added onto well beaten chemical pulp [65]. Thus, enhancement in tensile is expected to occur when NFC is added into filled paper, mechanical pulp and recycled pulp. Hii et al. [65] reported such observation that NFC gets adsorbed onto the filler and fibres and bridges the filler with the fibre network. The only major shortcoming of using NFC in papermaking is that it makes the drainage slower. Drainage plays a very much important role in papermaking as it directly relates to the efficiency of paper production. The slower the drainage time, the slower the paper will be produced. It is therefore very important to use retention agent at specific dosage to improve the adsorption of the nanofibres on the surface of the fibre, thus leading to improved dewatering [24, 68].

NFC has properties in between of dry strength additive such as starch and fines created from the action of beating as both of them lead to increase the bonded area [3]. This is achieved by creating a soft and thin layer on the fibre surface that helps to increase fibre bonding during drying and also by filling in voids and pores between fibres that also increase the bonded area [3].

#### Nanocellulose as Coating Material for Barrier Property

Nanocellulose can also be used as a coating material in packaging paper as it has good barrier properties. The benefit of using nanocellulose as a coating element is that dewatering problem is no longer an issue as it is added after the paper has been made. There are different approaches that can be employed to apply nanocellulose which include spraying, bar coating, size pressing and roll coating. The application of nanocellulose particularly nanofibrillated cellulose has been reported to increase oxygen barrier and oil resistance [69]. In one example, reduction in air permeability was observed from 69,000 to 4.8 and 660 to 0.2 nm Pa^−1^ for unbleached paper and grease proof paper, respectively [70], using the rod coater. Syverud and Stenius [71] applied varying amounts of NFC from 0 to 8% on softwood pulp and found that the barrier properties increased remarkably from 6.5 to 360 nm Pa^−1^ s^−1^. This is due to the reduced porosity caused by the increase in nanofibrils. Application of NFC and shellac was also attempted by Hult et al. [72] on paper and paperboard that results in the reduction of air permeability, oxygen transmission rate and water vapour transmission making it potential for barrier packaging.

Not only that, nanocellulose particularly nanofibrillated cellulose can be converted to free standing thin films or nanopapers. This nanopaper can be used as a substrate for electronic application as it is transparent and flexible [73].

#### Nanomaterial as Retention Agent for Property Improvement

Retention agents are added to the papermaking to improve the retention of functional chemicals on papers. Some nanomaterials have been tested in paper product such as the use of nanozeolite [74] and nanotitanium dioxide. Nanozeolite is used as dessicants in paper industry to absorb moisture and also functions to remove gas emission if used for specialty paper. The high surface area of nanozeolite consisting of voids and pores aids during such process. Nanotitanium oxide added in paper can form a paper with better dynamic elastic modulus compared to controlled sample [75].

#### Nanofiller Effect for Property Enhancement

The use of filler in the paper industry is mostly due to cost reduction as filler is usually cheaper than pulp itself. Besides being studied as a strength additive, nanocellulose can also be used as a filler. The addition of nanofibrillated cellulose can reduce the amount of wood pulp and increase the amount of filler, thus reducing production costs [76]. Furthermore, the paper produced has enhanced properties such as low porosity and high opacity. It was also reported that adding 2–10% nanofibrillated cellulose as a filler results in 50–90% strength increment (Future Markets Inc. 2012). Nanoclay can be used as an additive in papermaking to reduce gas permeability that can lead to longer shelf life of the paper. This is essential in packaging industry in which gas and water barriers play an important role in preventing food and drink spoilage.

Nanocalcium carbonate is used as a filler to improve light scattering. Modified precipitated calcium carbonate using nanostructured particles had a positive impact on the light scattering [77]. Precipitated calcium carbonated was coated with silicate and zinc sulphide nanoparticles. Wild et al. [78] reported a similar study in which nanoparticles coating was used at laboratory, pilot and mill trials. The study found that nanoparticle coating gives good printing quality, water permanence and dimensional stability. Nanozinc oxide added into paper furnish confers antibacterial property to paper. At the same time, optical properties such as brightness and whiteness as well as printability of the paper also improved upon addition of nanozinc oxide. Nanotitanium oxide has also been studied in combination with beta-cyclodextrin in coated paper [79]. It was found that the nanomaterial mixture had better degradation effect on xylene compared to paper coated with nanotitanium oxide only.

#### Nanomaterial as Sizing Agent for Property Improvement

A sizing agent is added onto papermaking to improve resistance of water/liquid penetration so that the paper is suitable for printing and writing purpose. The use of nanosilica can improve the optical properties and reduction in print-through up to 30%. Paper coated with nanosilica was found to produce better optical density, dimensional stability, print quality over uncoated paper [80–82].

### Wood Composites

Wood is a nature’s gift to humankind as it is a biodegradable and renewable material that can be used in many applications. However, the wood itself possesses several weaknesses such as delicare, non-flexible and non-durable because of termite attacks and others. The utilisation of wood fibres in producing wood composites has its own disadvantages as they have low bulk density, low thermal stability, high tendency to absorb moisture and susceptibility to biological degradation. Nanotechnology has been utilised in many sciences, and it can be used to improve the quality of many materials, including wood and wood-composites.

#### Nanocellulose as Reinforcement Material

The principle of using nanocellulose as a reinforcement material in the matrix material has led to many research works being conducted. By adding nanoscale cellulose, the nanocomposites possess outstanding properties in numerous ways, which could not be achieved by microcomposites [83]. These nanocellulose-reinforced composites are capable of replacing the conventional composites. Through appropriate modification of NCC, various functional nanomaterials with outstanding properties or significantly improved physical, chemical, biological, as well as electronic properties can be developed. Properties of nanocomposites depend on few factors such as the properties of matrix material, characteristics of nanocellulose, dispersion of nanocellulose in matrix material, and interfacial interactions between filler and matrix material [84].

**Fig. 2:**
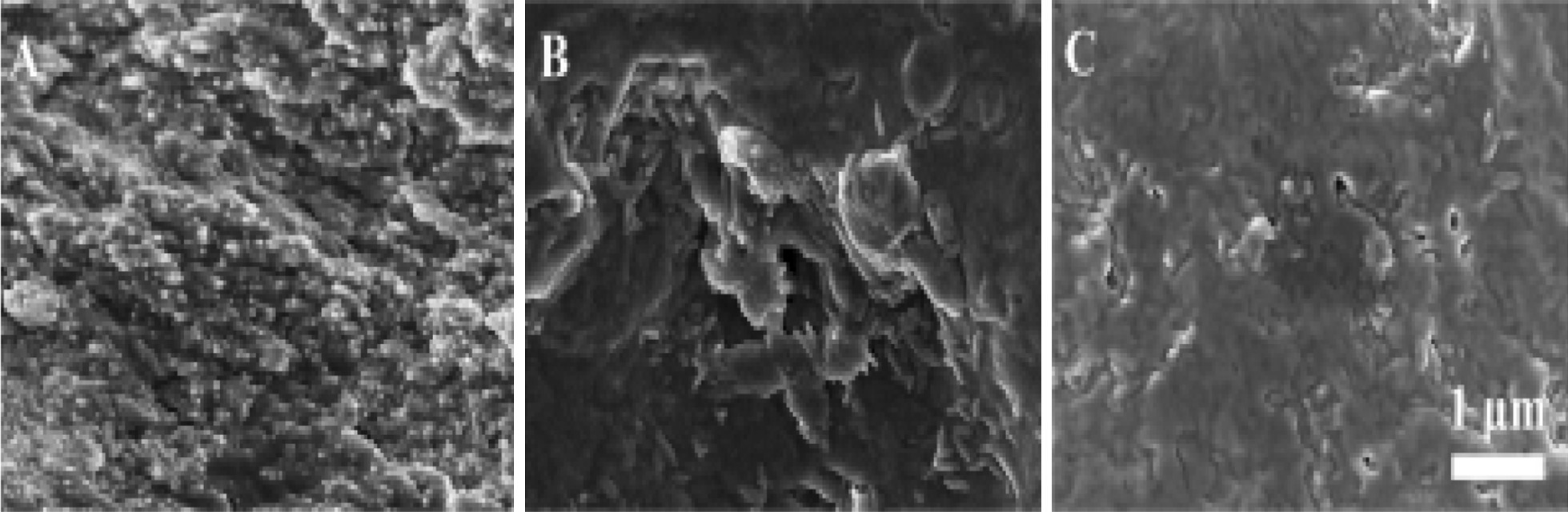
FE-SEM images for the fracture morphologies of **a** PHB/NCC-15, **b** PHB/acetylated NCC(II)-15, **c** PHB/acetylated NCC(IV)-15 nanocomposites. *Source*: Gan et al. [94]. Reproduced with permission of ACS

Nanocellulose as reinforcements into various types of matrix materials has been widely studied. Polymer nanocomposites consisting of natural or synthetic polymers reinforced with nanocellulose have established themselves as a promising class of materials. Improvement of the mechanical properties is the most common objective targeted when preparing these nanocellulose reinforced polymer [85–87]. Petroleum-based polymers are generally divided into thermoplastic and thermosetting types. The difference between thermoplastic and thermoset polymer is the bonding that holds their long-chain molecules, the former is held by weak van der Waals bonds, while the latter is by strong covalent bonds [88]. A variety of thermosets has been studied for use in nanocellulose composites. Epoxy resin, for example, has been utilised for advanced material products due to its excellent bonding properties, and good mechanical properties after curing (high modulus, low creep, and reasonable elevated temperature performance). However, it can easily fail under impact because of the highly cross-linked structure [89]. With the addition of the functionalised nanocellulose as the reinforcing material, the mechanical properties of epoxy-based cellulose nanocomposites have been enhanced significantly [90, 91]. An approach has been conducted to prepare epoxy-cellulose nanofibre composites with an oriented structure [92]. The process combined the ice-templating (or freeze-casting) method in order to prepare highly porous nanocellulose networks before being used as preforms for impregnation with a bioepoxy resin. The results showed that the elastic and storage moduli of the nanocomposites were better than those of pure epoxy in both testing directions, and the strength was improved in the longitudinal direction. Another most commonly used thermoset resin is unsaturated polyester (UP). Nanostructured UP biocomposites with nanocellulose content as high as 45 vol%, much higher than in any previous studies, were successfully processed and characterised [93]. The nanostructured nanocellulose network reinforcement strongly improves not only modulus and strength of UP but also ductility and toughness. Figure 2 shows the fracture morphologies of the hydrophobic polyester, poly(3-hydroxybutyrate-co-4-hydroxybutyrate) (PHB) either reinforced with 15 wt% NCC or 15 wt% acetylated NCC by using field-emission scanning electron microscopy (FE-SEM). The FE-SEM images clearly indicated that acetylated NCC was homogeneously dispersed into the PHB compared with NCC. The homogeneity has contributed to the strong interfacial interaction between the reinforcement material and the polymer matrix [94].

Table 2 illustrates the incorporation of nanocellulose into thermoplastic polymers such as PLA, PVA, starch, PU, PP and etcetera. The processes utilised to develop these nanocomposites include solvent exchange, aqueous dispersion, solution casting, grafting, core-back foam injection moulding, electro-spinning, coagulation and thermocompression, and in situ anionic ring-opening polymerisation reaction, among others. Most of the studies reported that the addition of nanocellulose in nanocomposites resulted in an improvement of mechanical properties (strength, stiffness, creep resistance, elasticity) thermal stability, barrier properties and even some indicated antibacterial and antioxidant functions of the nanocomposites, depending on the components of the nanocomposites used. Overall findings reported positive results on the reinforcement of nanocellulose in thermoplastic matrix which prompt and encourage more studies in producing greener and sustainable composite materials.

**Table 2:** Recently reported literature on the thermoplastic-based cellulose nanocomposites

Types of nanocellulose reinforcement	Thermoplastic matrix used	References
Modified NFC	Polylactic acid (PLA)	[95]
NCC		[96]
Modified NCC	Polyvinyl alcohol	[97, 98]
NCC	Starch	[99]
NFC	Polyurethane (PU)	[100]
NFC	Polypropylene (PP)	[101]
NFC	Polycaprolactone (PCL)	[102]
	Poly(methyl methacrylate) (PMMA)	[103]
NFC	Polyethylene (PE)	[104]
NCC	Polysulphone (PSU)	[105]
NCC	Chitosan	[106]
NCC	Poly(ethylene glycol) (PEG)	[107]
NFC	Polystyrene (PS)	[108]
NCC	Polyamide (PA)	[109]
NCC	Carboxymethyl cellulose (CMC)	[110]
NCC	Polyvinyl acetate	[111]
NCC	Polyhydroxybutyrate-co-valerate	[112]

Natural polymers are normally occurring in nature and can be extracted. Natural polymers are not thermoplastic, with only a few exceptions. However, chemical and physical modification techniques are able to trigger thermoplasticity in natural polymers from biomass resources such as cellulose, lignin, and chitin. Due to the increasing environmental consciousness and demand for green products, various natural polymers reinforced with nanocellulose have been used to produce bio-nanocomposites. There are very limited number of studies reporting the use of plant-derived nanocellulose in bone tissue regeneration. One of the studies [113] reported the preparation of a nanocomposite by synthesising (TEMPO)-oxidised nanofibrillated cellulose (TNFC) or nanocrystalline cellulose (NCC) with hydroxyapatite (HA). The composites were found to demonstrate better compression strengths, elastic moduli, and fracture toughness, in the range of outer and dense cortical bone, than the NCC-based ones. Furthermore, the composites did not induce cytotoxicity to human bone-derived osteoblast cells but rather improved their viability, making them promising for bone tissue regeneration in load-bearing applications.

Among other natural biopolymers used are sodium alginate, cellulose and proteins which have the characteristic properties such as biodegradable, biocompatible and low toxicity. Sodium alginate, for example, has been widely used as an excellent biomaterial in many fields such as tissue engineering, drug delivery, food packaging and biomedical applications. However, the poor mechanical strength and uncontrolled degradation properties limit its application. Several attempts have been conducted to overcome these problems by developing nanocomposite films with the incorporation of nanocellulose into the alginate matrix. It was found that the incorporation of NFC into the alginate matrix improved its water resistance and mechanical properties. Further investigation has shown that with ultrasonication treatment to facilitate the dispersion, TEMPO-mediated oxidised NCC has better efficiency in reinforcing alginate biopolymer compared to NFC. Similar concept of bioinspired synergistic reinforcing strategy in integrating nanocellulose into chitin, chitosan, soy protein isolate (SPI) and flaxseed gum matrix open a new path for constructing high-performance nanocomposites.

#### Nanoparticles for Wood-Based Panel Property Enhancement

Wood composites are generally described as a wide range of products having combination of wood elements held together by a binder. Among the advantages of wood composites are that they can be designed for specific qualities or performance requirements at different thicknesses, grades and sizes. Wood composites are manufactured to take advantage of the natural strength characteristics of wood (and sometimes results in a greater structural strength and stability than regular wood). On the other hand, wood composites also have disadvantages such that they require more primary energy to manufacture when compared to solid lumber. Hence, wood composites are not suitable for outdoor use as they can absorb water and are more prone to humidity-induced warping than solid woods. The adhesives used release toxic formaldehyde in the finished product. Nanotechnology can be utilised to improve the quality of wood-based composites to fulfil the increasing demand for existing products and for new products to be used in new applications.

The main drawbacks of wood are its susceptibility and biodegradability by microorganisms and also dimensional instability when subjected to varied moisture content. These are mostly due to the cell wall main polymers and their high abundance of hydroxyl groups (OH) [114]. Wood is naturally hygroscopic, and moisture absorption by wood materials is directly related to the exposed surface area. The addition of inorganic nanoparticles to wood composites has been reported to enhance the composites’ anti-microbial properties. Nanoparticles of zinc oxide (ZnO) exhibit good antimicrobial activity. These nanoparticles are added into melamine-urea formaldehyde (MUF) glue before being used for particleboard production [115]. The findings show that there were increments in bioresistance of the particleboards against the Gram-positive bacterium *Staphylococcus aureus*, the Gram-negative bacterium *Escherichia coli*, the molds *Aspergillus niger* and *Penicillium brevicompactum* as well as the brown-rot fungus *Coniophora puteana*. Silver nanoparticles which are well-known biocide additives also exhibited similar antibacterial and anti-mold efficiency effects when applied onto the melamine-laminated surfaces of particleboards [116]. The combination of nanocopper oxide and alkane surfactant was also confirmed to improve water and termite resistances of treated plywood specimens [117]. Modified starch-based adhesive was explored as another option to increase the decay resistance of particleboard. Particleboard bonded with modified PVA/oil palm starch added with nanosilicon oxide (SiO_2_) and boric acid was found to be more decay resistant than particleboard bonded with their native starch [118]. The addition of nano-SiO_2_ and boric acid as the water repellent and antifungal agents, respectively, have prevented the microorganism's activity in the final particleboard.

The manufacture of wood composite panels can be improvised by developing methods to shorten the cure time of the resin during hot-pressing, which could speed up production or improve overall quality of the board. Heat transfer which effects the pressing time of a wood-composite panel varies with thickness, press temperature, closing rate, and mat moisture distribution. The addition of ZnO nanoparticles increased the heat transfer at the centre of the particleboard during hot-pressing causing a greater degree of resin cure and improved the physico-mechanical properties [119]. High conductive nanoparticles such as multiwalled carbon nanotubes (CNTS) and aluminium oxide (Al_2_O_3_) were also proven to enhance thermal and mechanical properties of medium density fibreboard [120]. The study also reported that although activated carbon nanoparticles did not give any significant effect to physical and mechanical properties of the board, they have more accelerated effect on the curing of urea formaldehyde (UF) and reduction in the formaldehyde emission compared to the other two nanofillers.

In fabricating the wood composites, the adhesives play exceptionally significant role which affect the composites properties which include the mechanical properties, their ability to perform in wet conditions and their effects on the environment. Urea formaldehyde, melamine urea formaldehyde and phenol formaldehyde are commonly used in the wood composites industry. The utilisation of nanoparticles has led to improvements of the properties of adhesives. Many studies have been conducted to produce nanomaterial-reinforced wood composites with enhanced physical and mechanical performance and reduced formaldehyde emission. Nanoclays have been shown to be excellent fillers and reinforcement for the resin matrix, and significantly enhancing strength, toughness and other properties. The modification of UF adhesive using nanoclay particles for plywood fabricated with three forest species from fast-growth plantations: *Cordia alliodora*, *Gmelina arborea* and *Vochysia ferruginea* were reported by Muñoz and Moya [121]. It was determined that nanomodification of the resin with nanoclay at 0.75% improved the moduli of rupture and elasticity of the board. The effect of using nanoclay particles in PF adhesives significantly elevated mechanical properties of the adhesive in the bondline and contributed an increase in the macro-bonding strength of plywood [122]. Interestingly, transition metal ion-modified bentonite (TMI-BNT) nanoclay was used to covert crystalline UF resins to amorphous polymers by blocking the hydrogen bonds via in situ intercalation method [123]. This resulted in 56.4% increase in the adhesion strength and 48.3% reduction in the formaldehyde emission.

Other nanoparticles used to improvise the physical and mechanical properties of wood composites include nanowollastonite (NW) [124, 125] nano-ZnO [126], nano-SiO_2_, nano-Al_2_O_3_ [127], nanosilver [128] and nanocellulose (NCC and NFC). The NCC was utilised as filler for the adhesive, whereas NFC was applied as a binder to the formulation of the composite boards. Recently, it has been revealed that the addition of micro- and nanofibres of cellulose have advantageous effects on the properties of resin. Based on the investigations, it was found that the addition NCC significantly improved the mechanical properties of plywood in the amount of 10%/100 g of solid resin [129]. On the other hand, the physical properties of particleboard after adding the NCC to UF adhesives showed smooth surface, insignificant difference in the density and moisture content of the panels and only high value of nanocellulose content exhibited significantly higher in thickness swelling [130]. Meanwhile, particle boards panels manufactured using NFC as the bonding materials were shown to meet the industry requirements in terms of mechanical properties for low density grades [131]. For high density particleboard, it was estimated that the increased NFC ratio and higher pressure could improve internal bond properties. Even the nail and face screw withdrawal strength was found to be increased with the increase in NFC addition ratio and panel density [132, 133].

### Wood Coating

Forests are primarily or partially used for the production of wood and non-wood forest products. Non-wood forest products include bamboo, rattan, firewood, charcoal, damar, palm, etc. There is a huge demand for high quality wood, but the availability of wood from natural forest has been declining. Consequently, the search for non-wood resources as an alternative to wood has been accelerated. Due to its rapid growth property, bamboo has been developed into one of the most important non-wood forest products. Wood is a natural biologically self-assembled polymeric structure (cellulose, lignin, hemicellulose). It is one of the most versatile materials and has been used for centuries in the form of building and structures. However, wood is subjected to intense oxidative degradation processes such as photo-oxidation, chemical oxidation, thermal decomposition and photolysis reactions from the environment, including ultraviolet (UV) light, moisture, chemical pollutant and heat/cold variations [134]. Even non-wood materials like bamboo itself is a natural organic material which is rich in protein, carbohydrate and other nutrients and is prone to mildew, being eaten by moths and rotting. Hence, the final products of wood and non-wood products conventionally comprise additives which can be used as coatings for protection and aesthetic appearance improvement, preservatives for protection against fire and biological factors (fungi and insects).

Coating is a process of applying a layer to the substrate surface. Examples of common coatings applied to wood surfaces are varnishes, lacquers, and paints whose purpose can be both protective and decorative. The main components of the coatings determine their fundamental properties such as binders, pigments, solvents, fillers, and additives [135]. Each element contributes specific properties to the wood surface. The binder contributes to the adhesion of the pigment to the wood and creates a protective layer, while the pigment provides colour and form non-translucent surface layer. The solvents give necessary viscosity for coating application, and the addition of the fillers alters the colour strength and the gloss of the coating. As for the additives, they inhibit mould and decay, assist the drying process, improve the adhesion properties and control the finishing. However, there are weaknesses of coatings such as limited flexibility, strength loss, disproportionate adhesion between coating layer and substrate, inferior abrasion resistance and less durability.

Nanocoating has the capability to resolve these issues. Nanocoating is a process by which a thin layer of thickness about < 100 nm is deposited on the substrate for improving some properties or for imparting new functionality. The nanocoating can be used not only on nanomaterials but also on a bulkier material with an extremely thin layer coating without affecting the topography of the substrate surface. The application of nanocoating in wood and wood products is mainly focused on the improvement of durability, mechanical properties, fire resistance and UV absorption as well as decrease in water absorption. One approach to enhance the functionality and the end user value of nanocoating is the addition of nanoparticles [136]. These nanoparticles have very large surface-to-volume ratios due to their morphology, which allows them to interact intensively with their surroundings, and their nanosize ensures transparency is still sustained.

#### Nanoadditive for Durability Improvement

Nanocoatings are able to improve the durability of wood and non-wood products by utilising nanoparticles and nanodelivery systems that make the changes at the molecular level of the products. One of the aims of coatings is to prevent the growth of various microorganisms like fungi and bacteria. Nanosized particles of metal oxides, such as zinc oxide (ZnO) [137, 138], titanium oxide [138, 139] and cerium oxide (CeO_2_) [140] were reported to demonstrate strong antimicrobial properties. Studies have been conducted in the direct deposition of nanoparticles onto wood surfaces or direct functionalisation of wood surfaces with nanoparticles. ZnO nanoparticles were successfully fabricated on the surface of bamboo timber by a simple low-temperature wet chemical method based on sol–gel-prepared ZnO seed layers. The findings indicated that the treated bamboo timber had better resistance against *Aspergillus niger V. Tiegh* (A. niger) and *Penicillium citrinum Thom* (P. citrinum), but poor resistance against *Trichoderma viride Pers. ex Fr* (T. viride) [141]. Graphene also demonstrates superior ability to inhibit bacterial growth. Hence, the combination of utilising reduced graphene oxide and nano-ZnO to coat bamboo-based outdoor materials via a two-step dip-dry and hydrothermal process, resulting in the improvement of the mould resistance and antibacterial activity properties [142]. Similarly, the nanostructured ZnO using a hydrothermal process has also provided an effective protection of wood surfaces from biodeterioration [143].

Waterborne polyurethane [144] coatings (WPU) incorporated with nanocrystalline cellulose (NCC) and silver nanoparticles (AgNPs) were used to improve antibacterial property of wood board [145]. The AgNPs were known for their antimicrobial material but aggregated easily during the preparation process. Therefore, NCC was introduced to assist with the blending and dispersibility of AgNPs with WPU or other coatings. In addition, NCC was also a good reinforcing agent to improve the mechanical properties of nanocomposites. Mini emulsion polymerisation was also used to synthesise an acrylic latex coating containing AgNPs, which will limit the growth of black-stain fungi on the wood surfaces [146]. The study of the antibacterial effect of silver and zinc oxide nanoparticles in acrylic coatings applied during the treatment of commercial wooden composites such as particleboard and medium density fibreboard was conducted [147]. Ag and ZnO nanoparticles were partly more effective against the Gram-negative bacterium *Escherichia coli* compared to the Gram-positive bacterium *Staphylococcus aureus*.

#### Nanoadditive for Water Absorption Improvement

It is well known that wood is susceptible to water or moisture. This is due to the hydrophilic nature of the cell wall constituent polymer and its capillary-porous structure. The interaction between wood and water leads to biodegradation of wood, dimensional instability and accelerated weathering. Although there are conventional chemical modifications being used to improve the hydrophobicity of wood, the accessibility of water into wood is still not completely retarded [148]. Furthermore, the chemicals used in the treatment process are possibly hazardous. Hence, nanotechnology is used as an alternative approach for wood modification and functionalisation. The incorporation of nanoparticles into polymeric coatings is used to improve water absorption of wood surfaces. Two approaches are utilised to integrate the nanoparticles into the coatings, namely solution blending and in situ addition [149]. The first approach (solution blending) is when a solvent combines with the polymer before being dispersed onto the wood surfaces. This physical method can be applied through dipping, brushing and spraying [150]. Wu et al. [151] reported that a superhydrophobic coating was constructed on the surface of poplar wood with a contact angle of up to 158.4° through the waterborne UV lacquer product (WUV) which was modified by ZnO nanoparticles and stearic acid. Compared with WUV, the water resistance of zinc stearate/waterborne UV lacquer super-hydrophobic coating (ZnSt_2_/WUV) was stronger, which was conducive to prepare superhydrophobic coatings in an easy and environmentally friendly. Interestingly, a water-based varnish added with TiO_2_ nanoparticles was used to evaluate the finishing of nine tropical wood species [152]. It was found that the incorporation of TiO_2_ nanoparticles decreased the values of water absorption and after a year of weathering exposure, the varnish with no added TiO_2_ nanoparticles degraded completely, while the modified varnish film endured. Other examples of superhydrophobic wood coatings which were successfully prepared are lignin-coated nanocrystalline cellulose (L-NCC) particles/polyvinyl alcohol (PVA) composite paint system [153], UV-light curable methacrylic-siloxane-cellulose composite coatings [154], Fe^3+^-doped SiO_2_/TiO_2_ composite film [155] and polydimethylsiloxane (PDMS)/silica hybrid coating system [156].

The second approach is the in situ addition or a chemical process which involves compound addition directly to monomers and subsequent polymerisation. The nanoparticles are synthesised in situ by chemical reactions on the wood surface such as hydrothermal methods or solgel deposition. Gao et al. [157] applied a simple and effective method in preparing superhydrophobic conductive wood surface with super oil repellency using AgNPs modified by fluoroalkyl silane. The multifunctional coating could be commercialised for various applications, especially for self-cleaning and biomedical electronic devices. In another study, bamboo was treated using ZnO sol, and the ZnO nanosheet networks were grown hydrothermally onto the bamboo surface and subsequently modified with fluoroalkyl silane [158]. The successfully treated bamboo exhibited superior properties such as robust superhydrophobicity, stable repellency towards simulated acid rain, UV-resistant and fire-resistant. Similar superior properties were obtained when bamboo timber prepared by the hydrothermal deposition of anatase TiO_2_ nanoparticles and further modified with octadecyltrichlorosilane [159]. Superhydrophobic wood surfaces can also be prepared using approaches such as layer-by-layer [160] assembly of polyelectrolyte/TiO_2_ nanoparticles multi-layers and hydrophobic modified with perfluoroalkyltriethoxysilane (POTS) [161], spray coating of a waterborne perfluoroalkyl methacrylic copolymer (PMC)/TiO2 nanocomposites onto the PDMS pre-coated substrate [162] and a one-step hydrothermal process using tetrabutyltitanate (Ti(OC_4_H_9_)_4_, TBOT) and vinyltriethoxysilane (CH_2_CHSi(OC_2_H_5_)_3_, VTES) as a co-precursor [163]. Even a biomimetic approach as to produce a lotus-leaf-like SiO_2_ superhydrophobic bamboo surface based on soft lithography was successfully carried out [122].

#### Nanoadditive for Mechanical Properties Improvement

The inorganic particles integrated into organic polymers are commonly used in wood coatings to increase the mechanical properties. As fillers, the rigidity and hardness of the inorganic materials are combined effectively with the polymer’s processability. The inorganic particles when apply in micron size have disadvantages such as they reduce the flexibility of the material and decrease the transparency of the coating system [164]. The utilisation of the inorganic particles in nanosize increases the surface area and the ratio of the interfacial area, which subsequently influences the properties of the raw material [165]. Recent studies have investigated on using nanocellulose as a renewable reinforcement to develop a bio-based nanocomposite coating system with improved performance. Nanocellulose was surface modified due to the issue of incompatibility with the polymer matrix. The addition of TEMPO-oxidised cellulose nanofibres improved the mechanical properties of the WPU coating [144, 166]. In the case of the non-polar polymer matrix [167], nanocrystalline cellulose was modified by two methods, with acryloyl chloride or a cationic surfactant [167]. An increase in NCC loading level up to 2% increased hardness, elastic modulus, and tensile strength.

Nanosilica is another common nanoparticle that is applied for the improvement of mechanical properties. Among the advantages of using nanosilica are its high hardness and can easily be chemically modified to improve its compatibility with the polymer matrix. A recent study by Meng et al. [168] reported castor-oil-based waterborne acrylate (CWA)/SiO_2_ hybrid coatings with organic–inorganic covalent cross-linked network structures were prepared via solgel and thiol-ene reactions. The finding showed that beside the emulsions had good stability, the thermal and mechanical properties of the coating improved significantly at 10 wt% of SiO_2_. The improvement of mechanical properties with nanosilica addition was also described in other coating system such as waterborne nitrocellulose [169] and acrylate [170].

#### Nanoadditive for UV Absorption Improvement

The process of wood photodegradation begins directly after being exposed to solar light, and then the colour changes and progressive erosion of the wood surface occur. The UV radiation is capable of photochemically degrading the polymer structure components of wood (lignin, cellulose and hemicellulose) [171]. The photodegradation process usually results in reduced water resistance of wood and wood-based materials which lead to further biodegradation under outdoor exposure conditions. The intense damage to materials due to the UV component in solar radiation can be prevented by using light-stabilisation technologies, surface coatings or by replacing these materials with materials that are more resistant against UV radiation [172]. Nanoparticles can be utilised to improve the UV resistance for solvent, waterborne and UV coatings in order to protect the wood surfaces. Nanoparticles that contain functional coatings to achieve UV-blocking properties offer a high level of protection against UV without affecting the transparency of the surface. The small size of the nanoparticles gives a significant increase in effectiveness of blocking UV light compared to natural material due to their large surface area-to-volume ratio.

The use of UV radiation absorptive coatings serves to prevent lignin degradation from UV light. Among the nanoparticles used as UV absorbers are mainly TiO_2_ and ZnO. Wallenhorst et al. [173] reported a system composed of a Zn/ZnO coating and additional polyurethane sealing strongly reduced photodiscolouration of the wood surface and proved to be chemically stable. The combination of benzotriazole (BTZ) and ZnO nanoparticles was applied as the UV absorbers in acrylic-based bamboo exterior coatings [174]. Strong synergistic effects were detected in the BTZ–ZnO coatings, especially for the 2:1 ratio formulation. The coating system provided high resistance to photodegradation and effectively inhibited photodiscolouration of the bamboo substrates. Another mixture of benzotriazoles, hindered amine light stabilizers (HALS), and ZnO nanoparticles in thick-film waterborne acrylic coating also gave the most positive effect in UV protective surface modification when applied to oak wood [175]. The mixture of benzotriazoles, HALS and both TiO_2_ and ZnO nanoparticles was confirmed as one of the most effective treatments for colour stabilisation of wood due to UV and VIS spectrums. It was reported that wood specimens coated with rutile TiO_2_ and a mixture of methyltrimethoxysilane and hexadecyltrimethoxysilane showed superior weathering performance and improved resistance to surface colour change and weight loss [176]. The TiO_2_ coating also was found to apparently enhance the colour stability of wood during UV light irradiation without water spray. However, the adjacent wood surface degraded because of the photocatalytic activity of TiO_2_ [177].

#### Nanoadditive for Fire Retardancy Improvement

The flammability of wood and non-wood products has restricted utilisation, with fire safety being a major concern for the various applications. To overcome the inherent deficiencies and use of wood and non-wood in a safe manner, the flame retardant properties need serious consideration. Nanoparticles have recently been used to produce the nanocomposites for the improvement in fire retardant properties. The utilisation of nanoparticles, either alone or in combination with conventional fire retardants, serves to reduce the ignitability of wood. The nanosize and high surface area of nanomaterials make them more effective at low concentrations than other conventional compounds which are an enormous advantage industrially and economically. The surface modification is necessary for nanoparticles to achieve better compatible and homogeneous dispersion. The TiO_2_ coated wood was found to be capable in reducing the flammability of the wood and the spreading of the flame, as compared to the uncoated sample [178]. The ZnO–TiO_2_-layered double-nanostructures had been synthesised on a bamboo substrate [179]. The findings showed that the oxygen index increased from 25.6 to 30.2% after being covered with a ZnO–TiO_2_ coating, which revealed a significant enhancement of its flame retardant property. Layered double hydroxides [180] can absorb a large amount of heat, dilute the concentration of flammable gas, and absorb harmful acid gases during the decomposition process; therefore, it is an excellent flame retardant. Yao et al. [180] applied nanomagnesium aluminium layered double hydroxide (Mg–Al LDH) to bamboo in an in situ one-step process and found that the total heat release and total smoke production were reduced by 33.3% and 88.9%, respectively, compared to those of samples without Mg–Al LDH. Wang et al. [181] introduced zinc-aluminum layered double hydroxide (Zn–Al LDH) nanostructures to wood and found that the peak heat release rate (PHRR) and total smoke production were reduced by 55% and 47%, respectively, compared to those of the pristine wood [181]. Nanostructured carbon materials such as graphene was also proven to have a great potential to be used as an effective fire retardant in wood and wood-composite materials for surface protection against fire [182].

### Wood Durability

Wood is such a versatile material that finds its use in various fields like construction, furniture and artwork [183–185]. Wood is applied as a construction material due to its high strength-to-weight ratio, eco-friendly characteristic, aesthetic appearance and biodegradability feature. Unfortunately, wood is very sensitive to biological attacks, especially by decay fungi and insects [186, 187]. Wood also gets affected by exposure to UV-radiation, fire and moisture [188]. Moisture can cause wood warping, cracking and dimensional instability. The wood degradation can cause aesthetical and internal structural damages. The degradation of wood leads to immeasurable losses each year. Besides, the growth of decay fungi on wood structural will trigger health problem to human including allergies, respiratory symptoms and asthma especially after prolonged indoor exposures [189]. Therefore, the associated issues related to wood cannot be simply overlooked. Preservation of wood using chemical is the effective way to protect and prolong the service life of wood.

Over the last decades, many chemical preservatives have been developed and used to protect wood against biodegradation agents [188, 190]. Unfortunately, most of these chemical preservatives may pose serious effects to human, living organisms and environment due to their accumulation in soil and ecosystems [191]. Chromated copper arsenate (CCA) is one of the chemical preservatives widely used since the middle 1930s and effectively protects wood against decaying fungi, termite and insect borer. However, CCA was shown to be toxic to human and environment [192–194]. A similar issue is also faced by another chemical, i.e. pentachlorophenol (PCP). It was considered to be hazardous to human health; thus, its production and uses were banned in many countries [195].

In response to these issues, a new series of chemical preservatives claimed as safe and less-polluting are being introduced. These chemical preservatives can be obtained from plant extract or produced synthetically and have plant bioactive compound properties. For instance, pyrethroids, derived from pyrethrum flower (*Chrysanthemum cinerariifolium*), have a potent insecticidal activity and can now be produced synthetically. Unfortunately, these chemicals were found easily degradable by light and temperature, and have narrow efficiency spectrum [196]. Other limitation of organic pesticides is that most of them dissolve in organic solvents and cannot be formulated as water-based wood preservatives. Therefore, smart and intelligent organic pesticides delivery system is crucial. Through a smart delivery system, biocides can be delivered in a controlled and targeted manner. This will reduce hazards to human and environment [17].

Wood treatment via nanotechnology method can improve wood durability against biodegradation agents and weather [14, 197, 198]. The main advantage of applying nanoproducts to wood is its unique ability to penetrate deeply in wood structure [14, 199], thus improving durability properties that result in a long life time service. On the other hand, complete penetration with uniform distribution of nanoproducts can also be achieved [200]. To date, many ready-to-use nanoproducts (nanopreservatives) for wood protection are available in the market, while some of them are still in the research and development stage. Generally, nanoproducts used to protect wood can be classified into two types, namely nanocapsules and nanomaterials. Nanocapsule refers to the pesticides embedded in a polymeric nanocarrier, while nanomaterial is nanosized metals which can be directly impregnated into wood [201].

#### Biocide Enhancement Property via Nanoencapsulation

Encapsulation of pesticides into polymeric nanocarriers is one of the promising nanotechnology techniques in improving the impregnation of wood with pesticides. This technique is designed to increase the solubility of poorly water-soluble pesticides and to release pesticides in a slow manner [202]. Encapsulation allows those low solubility pesticides to disperse easily in solid polymeric nanoparticles. The polymer can then be suspended in water and applied to wood via conventional water-based treatments [149]. Encapsulation is also able to protect the hydrophilic active ingredient from excessive leaching [19]. Due to the small diameter of capsule, it can be easily incorporated and penetrated into the cell wall of the wood. This accordingly improves the durability of treated wood against biodegradation agents. Not only this technology can deliver pesticides safely, it can also prolong the pesticide lifespan, resulting in extended protection for the wood [203].

Encapsulation of pesticides is a bottom-up approach. It can be carried out through several techniques such as nanoprecipitation [17, 204–206], emulsion-diffusion [207] and double emulsification [208] (Table 3). The materials used as polymeric nanocarriers can be derived from natural polymer, synthetic polymer or combination of both [209]. For instance, natural polymer includes cellulose, starch, alginates, silica and halloysite. The synthetic polymer usually used as polymeric nanocarriers includes poly(vinyl acetate), poly(methyl methacrylate), poly(lactic acid), etc. (Table 3).

**Table 3:** Pesticide nanocapsules prepared using natural and synthetic polymer

Polymer	Co-polymer	Pesticides	Encapsulation method	References
Alginate	Chitosan	Acetamiprid	Ionic pregelation and polyelectrolyte complexation	[210]
Alginate	Poly(vinyl alcohol)	Imidachoprid	Emulsion cross-linking	[191]
Chitosan	Poly(methyl methacrylate)	Tebuconazole	Amphiphilic self-assembling	[211]
Starch	Acrylic acid, methyl methacrylate	Carbendazem	Solution polymerisation	[212]
Silica	Cellulose nanofibrils	Tebuconazole	–	[213]
Polyethyleneimine	–	Sodium benzoate	Interfacial polymerisation	[214]
Polyvinylpyridine	Styrene	Tebuconazole and chlorothalonil	Impregnation method	[18]
Hollow silica	–	Tebuconazole	Miniemulsion	[215]

A previous study by Liu et al. [18, 216] successfully encapsulated tebuconazole and chlorothalonil into polyvinylpyridine and polyvinylpyridine-co-styrene using the impregnation method. The particle size of capsules obtained was between 100 and 250 nm. They impregnated a suspension of the capsules into sapwood of southern yellow pine and birch using conventional pressure treatments. The treated wood was then exposed to brown-rot (*Gloeophyllum trabeum*) and white-rot (*Trametes versicolour*) fungi for 55 days. The results showed great resistance against both decay fungi.

Salma et al. [17] used the nanoprecipitation technique to encapsulate tebuconazole. The polymer capsules containing tebuconazole were prepared from amphiphilic copolymers of gelatine grafted with methyl methacrylate with the size diameters ranging from 200 to 400 nm or 10 to 100 nm depending on core/polymer shell mass ratio. The encapsulated tebuconazole was reported to be able to preserve wood against a brown rot fungus. On the other hand, the formulation system developed by Salma et al. [17] is flexible and can be easily modified using copolymerisation of other acrylic monomers like hydroxyethyl methacrylate. This indicates that tebuconazole release rate can be tailor-made. However, the disadvantage of this nanocapsules is that they are prone to aggregation, which reduces the delivery efficiency of the nanocapsules into the wood.

Can et al. [217] successfully encapsulated nano silver into polystyrene-soybean co-polymer. In their study, the Scots pine was impregnated with the capsules and tested against white-rot fungi (*Trametes versicolor*). The finding obtained from the study indicates that the soybean oil, polystyrene and nanosilver played important roles in the synergistic effect of increasing the decay resistance of Scots pine.

#### Impregnation of Metallic Nanoparticles for Durability Enhancement

Metallic nanoparticles have been used to protect wood against biodegradation agents and weathering since decades ago [197, 218, 219]. Nanoparticles offer better characteristics than their bulk form, mainly because of their reduced size that leads to high specific surface area-to-volume ratio, uniform size distribution and good stability. Due to its very small size, nanoparticles can penetrate deeply and uniformly into the wood pores leading to a protection of wood [197, 220]. In addition, dispersion stability is improved because of the size and also by addition of surfactant [221]. Dispersion stability coupled with small particle size may greatly improve the following aspects: (1) preservative penetration, (2) treatability of wood, (3) stability of finishes and coating products, (4) low viscosity, and (5) non-leachability [222, 223]. Besides, it can enhance compatibility with binders thereby being able to increase the affinity with wood polymers [197, 224].

**Fig. 3:**
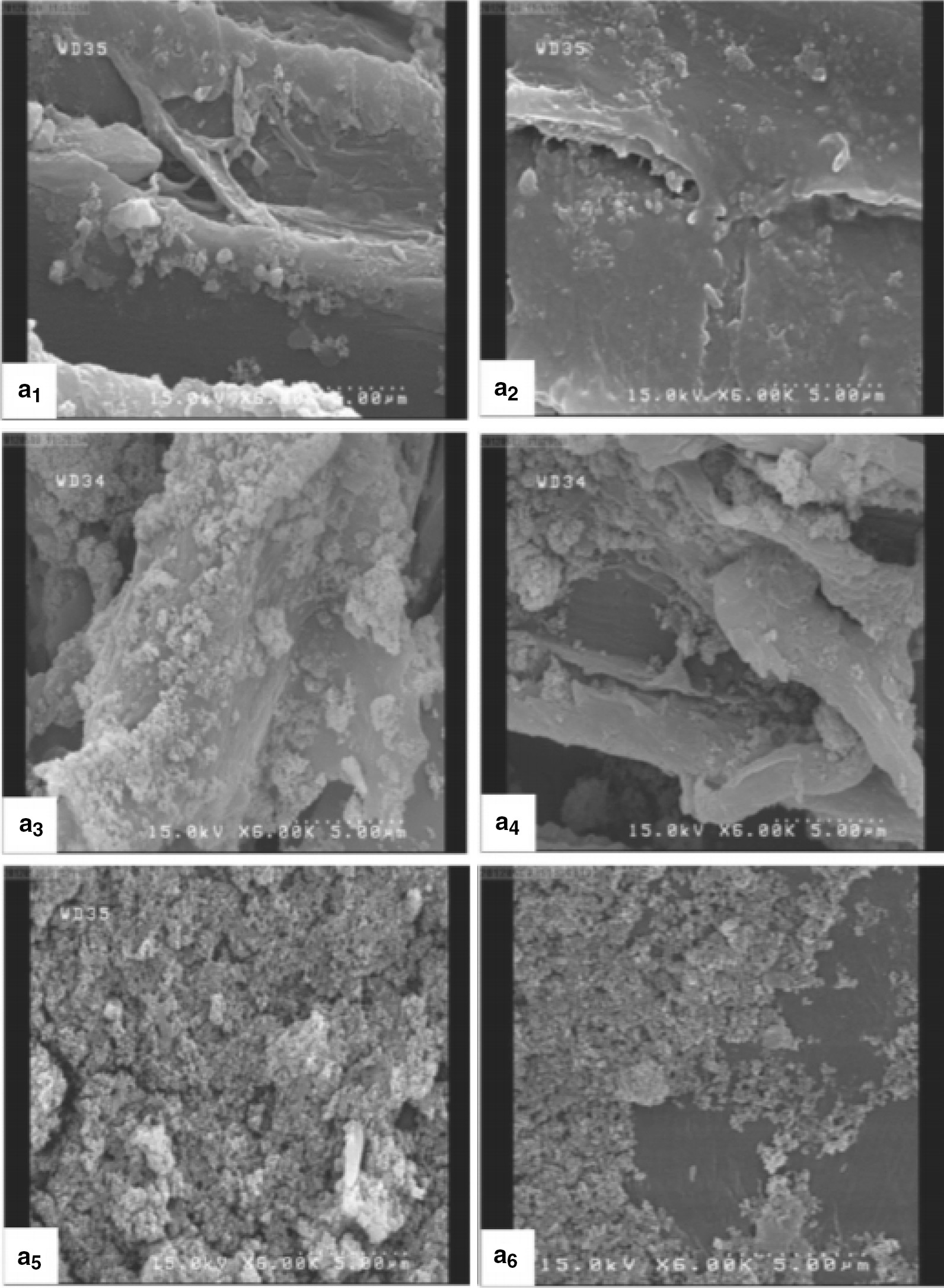
SEM images taken from the tangential section of nano-TiO_2_ wood treated samples: the 0% moisture content (MC)/0.5% concentration (a1), the 25% MC/0.5% concentration (A2), the 0% MC/1% concentration (A3), the 25% MC/1% concentration (A4), the 0% MC/1.5% concentration (A5) and (25% MC/1.5% concentration. *Source*: Afrouzi et al. [227]

Metallic nanoparticle can be prepared by altering the particulate size of metal via chemical reactions, mechanical treatment, heating or refluxing. To date, a lot of nanoparticles have been used for wood protection. Metallic nanoparticles mainly copper, silver, boron and zinc exhibited a good performance against white-rot and brown-rot but less efficient against mould [219]. Titanium dioxide (TiO_2_) is another nanoparticle that has a good potential to be used as wood preservative. The potential of TiO_2_ is related to its antibacterial and antifungal [14, 225] and UV-resistant properties [225, 226]. However, the study on wood treated with TiO_2_ is still under preliminary investigation [14, 225]. Table 4 lists the works on the utilisation of TiO_2_ for wood preservation. The distribution of TiO_2_ in wood was studied by Mohammadnia-Afrouzi et al. (Fig. 3).

**Table 4:** List of study on utilisation of titanium dioxide for wood preservation

Research	Findings	References
Study the effect of moisture content (0% and 25%) on the TiO_2_ nanoparticles retention and distribution in cottonwood	Better impregnation and uniform distribution of TiO_2_ in cottonwood was observed for the 0% moisture content sample compared than the 25%	[227]
Developed TiO_2_-Oligoaldaramide core–shell as efficient water soluble compounds systems for wood preservation	Wood treated with TiO_2_-Oligoaldaramine shows a good efficacy against attack by *Trametes versicolor*	[225]
Study the antifungal properties of TiO_2_ and ZnO nanoparticles in polyvinyl butyral	Treatment with combination of 2% TiO_2_ and ZnO nanoparticles in 5% polyvinyl butyral provided antifungal effects against *Trametes versicolor*	[228]
Study the UV resistant property of wood treated with zinc oxide (ZnO), cerium oxide (CeO_2_) and titanium oxide (TiO_2_) nanoparticles. All nanoparticles were dispersed in propylene glycol (PPG)	The nanoparticles restricted lignin degradation by UV radiation. Increasing the concentration of nanoparticles from 1 to 2.5% significantly enhanced UV resistance	[229]

Copper is an essential biocide for wood protection. However, copper alone fails to protect wood against copper-tolerant wood destroying fungi. Copper nanoparticles is a new generation of wood preservative-based copper. The use of copper nanoparticles instead of conventional copper shows improved durability of wood against decay fungi [230]. This shows that copper nanoparticles can be used to protect wood without the presence of chromium and arsenic [230].

Cristea et al. [15] studied the effects of the addition of ZnO nanoparticles and silver nanoparticles into exterior wood coatings. The purpose of the study is to improve the durability and wood protection through UV shielding. Besides providing an efficient protection against UV, the mechanical properties of wood such as hardness, adhesion strength, the abrasion resistance and the barrier effect for water vapor diffusion were slightly improved.

The mixture of ZnO nanoparticles with silver nanoparticles was able to protect wood from weathering problem such as UV rays [231]. They impregnated sapwood of cottonwood using three different concentrations of mixture by full-cell process. The samples were then exposed to natural weathering. The colour changes of treated wood samples were measured using spectrophotometer. The wood treated with ZnO nanoparticles alone was used as a control. The finding indicates that wood treated with the mixture of ZnO nanoparticle and silver nanoparticles has the lowest colour changes compared to the samples treated with each metallic nanoparticles.

In another study, Mantanis et al. [197] treated black pine wood with ZnO, zinc borate and copper oxide nanoparticles under vacuum. They used acrylic emulsion to force the metallic nanoparticles into the wood structure to avoid leaching. The durability of treated wood against mould decay fungi and subterranean termites was evaluated. Results showed that wood treated with zinc borate slightly inhibited the mold, while the other metallic nanoparticles did not exhibit mould. A similar finding was reported by Terzi et al. [232] that ZnO nanoparticles did not exhibit the mould growth on wood specimens. However, all metallic nanoparticles significantly inhibited white-rot fungi and termite.

Another study shows that metallic nanoparticles are able to improve fire retardant properties of wood. Francĕs et al. [16] studied the effect of SiO_2_, TiO_2_ and ZnO_2_ infiltrated into pine veneers. They reported that veneer treated using 3 wt% of SiO_2_ was most effective to improve the fire retardant behaviour.

Despite the remarkable advantages of nanotechnology in the wood preservation sector as discussed above, the fundamental understanding on synthesis, processing and characterisation of nanocapsules and metallic nanoparticles for wood protection still needs to be improved. The most interesting characteristics need to be considered during the design and development of nanocapsules or metallic nanoparticles for wood protection are as listed in Table 5.

**Table 5:** Characteristics of nanocapsules and metallic nanomaterials for wood protection

Purpose	Properties of nanomaterials
Long-term outdoor performance	Light stability (UV resistance)
	High transparency with ideal accentuation of the wood grain for highlighted the natural beauty of the wood
	Good mechanical and chemical resistances
Long-term durability	Toxic to bio-deteriorating agents but safe for human and environment
	Controlled release properties
Long-term dirt-repelling	Self-cleaning
	Water repellent

### Potential of Nanocellulose-Based Material in Energy Sector

Energy is an important resource that has a strong correlation between economic growth and development [233]. Today, the main energy sources are from fossil fuels and hydroelectric sources, which are very harmful to environment because they can cause climate change and global warning as well as ozone layer depletion, pollution, greenhouse gases emission and ecological destruction [234–236]. About 80% of carbon dioxide (CO_2_) emissions in the world are from energy sector and technology advancement is required to develop sustainable renewable energy resources to reduce the CO_2_ emissions as well as to overcome the global warming impact on life and health in line with the needs of accelerating technology development [234, 237].

In order to minimise the environmental effects, a sustainable and low-cost energy efficient carbon-based material has been explored as a potential to replace some conventional materials in the fabrication of energy devices. One of the natural carbon-based materials is cellulose which is the most promising natural polymer with many usages, including energy [237].

Nanotechnology is one of the advanced technologies that have the potential and prospect to fulfil the demand to create clean and green energy. Developing this new material in nanoscale enables new application and its interaction with current energy technology that would revolutionise the energy field from usage to supply, conversion to storage and transmission to distribution [238]. By adapting this nanotechnology, it will have high impact on the development of clean and green energy and benefits the environment and natural resources [239].

According to Serrano et al. [234] and Hut et al. [235], most promising application of nanotechnology for energy conversion is mainly focused on solar energy, conductive materials, solar hydrogen, fuel cells, batteries, power generation and energy devices. Understanding the structural and morphological properties of nanomaterial is essential to obtain the proficiency and sustainability for many applications. The greatest application of nanotechnology in energy generation is solar energy using photovoltaic (PV) cells which focuses on harnessing efficiency [233]. Consumption of energy generated from this solar cell using natural resources will reduce the usage of fossil fuel and decrease the pollution towards creating environmentally friendly and green energy [239]. In addition, the development of nano devices using solar cell could improve the existing materials efficiency as well as reducing manufacturing cost that might increase the economic growth [236].

Nanotechnology has been used in various applications to improve the environment, to solve humanity problems and to produce more efficient and cost-effective energy, such as generating less pollution during products manufacturing, producing solar and fuel cells at a competitive cost, hydrogen production, cleaning up organic chemicals polluting groundwater and overcoming the problem of energy sufficiency, climate change and diseases as well as to reduce the dependency on non-renewable energy sources [233, 235, 240].

#### Nanocellulose-Based Material for Solar Energy

Solar energy is available in various parts of the world and can be captured from the sun with 15,000 times more energy yearly. This energy source can be used in different ways: photovoltaic (PV) technology, solar thermal systems, artificial photosynthesis, passive solar technologies and biomass technology, which are used to produce electricity, steam or biofuels [234, 235].

In future, nanotechnology might contribute to develop an effective and low-cost system for production, storage, and transporting of energy [235]. According to Serrano et al. [234] and Hut et al. [235], current photovoltaic (PV) market is based on silicon wafer-based solar cells (first generation) and thin film layers of semiconductor materials (second generation). Current drawback of using solar cells is the cost of manufacturing mainly on the high cost of conventional PV cells with poor energy absorption efficiency (less than 40%) [233].

Nanocellulose shows a good potential to be used in the solar energy system due to its renewability, biodegradability, biocompatibility, broad modification capacity, adaptability and versatile morphology [241, 242]. Low cost, flexible and porous substrate of cellulose could be used to produce solar cells. Nanofibrillated cellulose (NFC) with size as low as 4 nm could become the excellent candidate for production of ultrathin paper for use in solar cell component to store the energy [235]. Klochko et al. [243] used nanocellulose from biomass for the development of biodegradable eco-friendly flexible thin film as a thermoelectric material. The thin film was used to convert low-grade waste heat from sun radiation into electricity at near-room temperature.

#### Nanocellulose-Based Conductive Materials

Conductive materials allow the flow of electrical current which is needed in the fabrication of energy devices. There are many types of conductive materials such as conductive polymers and conductive carbon materials (e.g. carbon nanotubes, graphene, and carbon black) and metallic particle with different levels of conductivity. These conductive materials can be combined with nanocellulose to form novel composites. The process of production conductive nanocellulose is shown in Fig. 2. There are two major strategies involved in nanocellulose based conductive hybrids fabrication process; one is coating of conductive materials layer on the surface of nanocellulose substrates, and another one is mixing the conductive materials inside the nanocellulose substrate to make composite [237]. Conductive polymers are an alternative to metallic materials because of their good electrochemical performance, light in weight and low cost. One of the most promising conductive polymers is polyaniline (PANI) because of its simple route of synthesis, controllable conductivity and high specific capacitance [244].

Nanocellulose-based conductive materials are developed for supercapacitors and energy storage device applications using various types of method such as in situ polymerisation, doping, coating, inkjet printing and in situ depositing [68, 244–246]. Modification of the existing supercapacitor by adding nanocomponents has increased its ability to store large amount of energy with longer time of supply [238].

Besides that, nanocellulose-based composite membrane electrodes can be developed via in situ polymerisation of nanocellulose using conductive components via a simple filtration unit (Fig. 3). A well-mixed conductive materials/nanocellulose composite membrane is left on the filter after the liquid has passed through the filter and air-dried composite membrane can be peeled off from the filter membrane for further use as supercapacitors [244].

#### Nanocellulose for Energy Storage

The potential application of nanocellulose for energy storage application has gained much attention recently. This is due to its nanoscale dimension, high surface area-to-volume ratio, and rich with hydroxyl group, which make their surface chemistry easily modifiable for composite processing. The most important aspect in energy storage is to develop nanocellulose with conductivity and flexibility properties. It can be achieved by adding conducting polymers such as polyaniline and polypyrrole. For example, the nanocellulose/polyaniline composite film is widely used as paper based sensors, flexible electrode, and conducting adhesive [68, 247–249]. Razaq et al. [250] manufactured electrodes from the composite of nanocellulose/polypyrrole and carbon filament for paper based energy storage devices. Wang et al. [248] reported that their devices which developed using composite of nanocellulose/polypyrrole provide high charge and discharge rate capabilities, high cell capacitances, and cycling performance.

##### Nanocellulose-Based Materials for Lithium and Vanadium Battery

High demand on flexible portable electronic devices recently such as smart phone, electrical vehicles, laptops, and even the grid energy storage causes increasing demand on rechargeable lithium-ion battery (LIBs) [251] and supercapacitors [252]. LIBs are one of the most ideal energy storage candidates for electronic devices, due to their high energy density, moderate power density and cycle stability. In LIBs, electrolyte is important for lithium-ion (Li^+^) transfer between anode and cathode. Organic liquid electrolyte is used for LIBs system, but it can pose tremendous safety concern due to the high toxicity and flammability. Solid-state electrolyte has become of interest because it demonstrates obvious advantages of low flammability and low toxicity [253, 254]. According to Janek et al. [255], solid-state electrolyte is classified as solid polymer electrolyte (SPE) and inorganic solid electrolyte (ISE). However, SPE offers the advantages of easy processing and flexibility [255].

Qin et al. [256] developed SPE by incorporating polyethylene oxide (PEO) with nanofibrillated aerogel and bis(trifluoromethanesulphonyl)imide lithium salt (LiTFSI). The results showed that the ionic conductivity properties of SPE were significantly enhanced due to the negatively charged nanofibrillated cellulose. The results also proved that the fabricated SPE is electrochemically stable, mechanically robust and thermally stable as well as flexible, expected for use in flexible electronic devices.

Nair el at. [257] fabricated nanocellulose-laden composite polymer electrolyte for high performing lithium-sulphur batteries using a thermally induced polymerisation method. The composite polymer electrolyte demonstrates excellent ionic conductivity, thermal stability up to more than 200 °C and stable interface towards lithium. The electrolyte also has stable cycling profiles which are attributed to significant reduction of the migration of polysulphide towards anode by entrapment of nanocellulose in the polymer matrix.

Another study was carried out by Zhang et al. [258] on robust proton exchange membrane developed using sulphonated poly(ether sulphone) reinforced by core–shell nanocellulose for vanadium redox flow batteries (VRFBs). It was found that with the incorporation of silica–encapsulated nanocellulose, the proton exchange membrane exhibits outstanding mechanical strength of 54.5 MPa and high energy efficiency above 82% at 100 mA cm^−2^, which is stable during 200 charge–discharge cycles. Proton exchange membrane is one of the key components in VRFBs. It functions as the separator to avoid vanadium ions crossover. It also acts as proton conductors that contribute to high voltage efficiency in VRFBs [259].

##### Nanocellulose-Based Materials for Flexible Supercapacitor

Supercapacitor or known as electrochemical double-layer capacitor or ultracapacitor is another versatile energy storage system that has gained the attention of researchers worldwide [260]. With the high power density, long life cycle, simple principles, low maintenance, portability, stable performance and fast charge/discharge rate make supercapacitors capable of filling the gap between batteries and conventional capacitors [260]. These properties offer a promising approach to meet the growing power demands.

Electrode is a very important component in supercapacitor. It requires good electrochemical performance and flexibility especially for preparing a high-performance flexible supercapacitor. Him et al. [261] and Zhe et al. [262] found that graphene and nanocellulose are excellent flexible electrode materials for supercapacitors. Nanocellulose has been used as a substrate material because of its good biodegradability, mechanical strength, flexibility, and chemical reactivity. The porous structure and hydrophilicity of nanocellulose can facilitate the attachment of other materials for example graphene in their fibrous network structure [263]. At the same time, the abundance of hydroxyl groups on the nanocellulose surface enables the interaction of nanocellulose with other polymer to form strong composites [264].

Khosrozadeh et al. [265] developed an electrode for supercapacitor using nanocellulose-based polyaniline/graphene/silver nanowire composite and after applying it for 2400 cycles, at a current density of 1.6 A/g, the supercapacitor showed a power density, energy density and capacitance of 108%, 98% and 84%, respectively. This shows that the electrode has an excellent cyclic stability and good mechanical flexibility. On the other hand, Ma et al. [266] developed an electrode using bacteria cellulose/polypyrrole coated with graphene. The prepared electrode has a good mechanical flexibility in which it can bend at any angle. The area capacitance and energy density can reach 790 mF cm^−2^ and 0.11 mWh cm^−2^, respectively, when assembled into symmetric supercapacitors. The nanocellulose-graphene electrode can be fabricated using chemical cross-linking or physical cross-linking method [267, 268]. Nanocellulose as an electrode component of supercapacitor also plays the role of internal electrolyte reservoir. This is because nanocellulose can provide an effective way for ion transport. The high pore structure and hydrophilic properties of nanocellulose make it easier to transport electrolyte ion [261].

#### Nanocellulose-Based Paper for Electronic Devices

Nanocellulose-based paper is a green substrate that can be used for electronic and optoelectronic devices. Currently, commercial paper has relatively rough surface and weak mechanical properties which can be quite problematic for electronic device fabrication [269]. Most of the electronic device’s fabrication use non-biodegradable and non-recyclable component such as plastics, glasses and silicones as substrates [270]. Formation of nanocellulose-based paper from NFC via simple filtration method can produce mechanically strong and low coefficient thermal expansion paper as an alternative to commercial paper [6, 269]. According to Li and Lee [270], transparent nanocellulose-based paper for electronic devices has been designed and being applied for electrochromic, touch sensor, solar cells, transistors, organic light-emitting diodes (OLEDs), gravure printing proofer and radio-frequency identification (RFID).

**Fig. 4:**
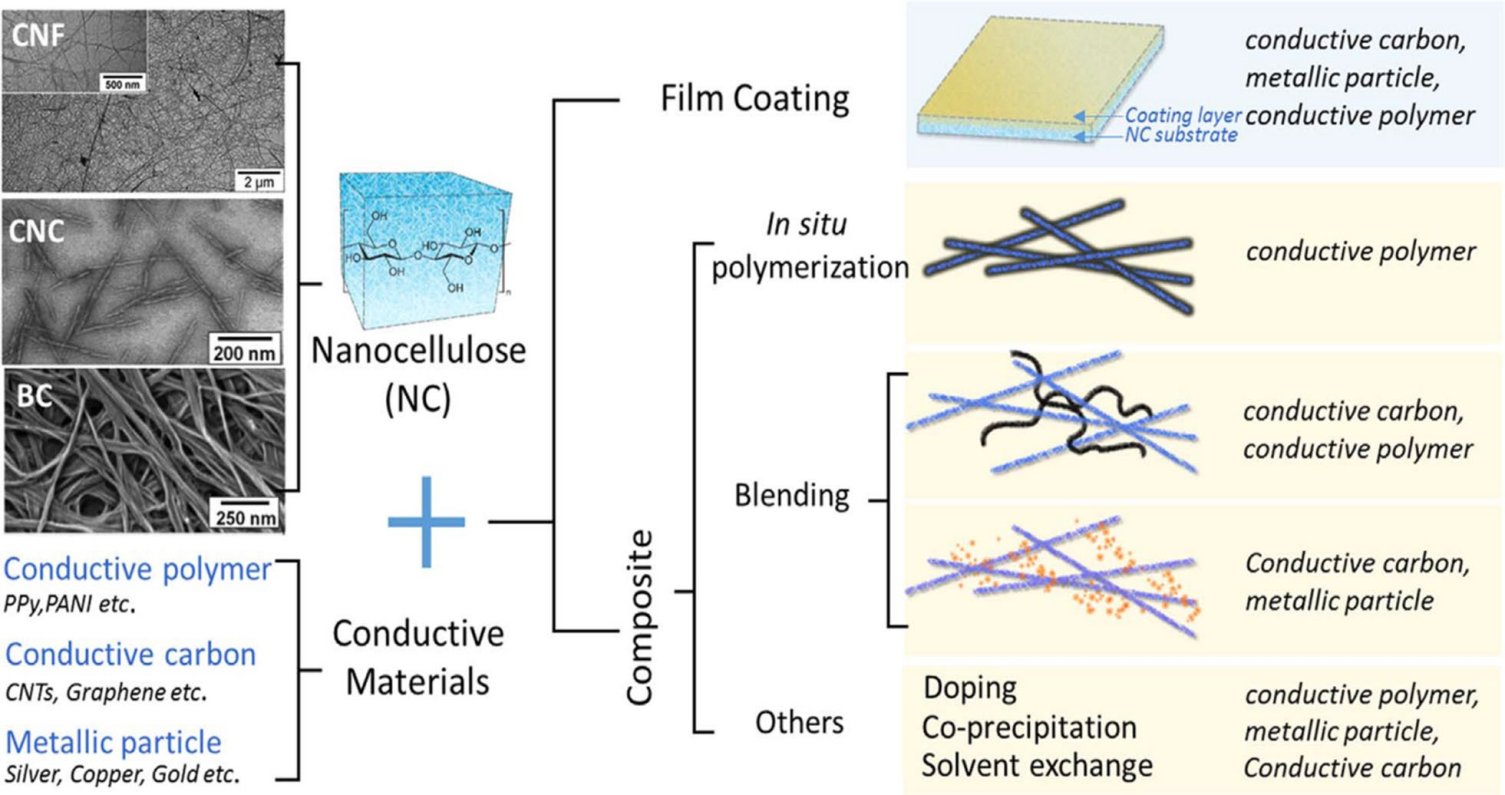
Schematic illustration of the generalised fabrication routes to nanocellulose-based conductive hybrid. *Source*: Du et al. [237]. Reproduced with permission of Elsevier

One of the applications of transparent nanocellulose paper is for the production of flexible electronics materials through printing circuit directly on the surface of substrates via coating or thermal deposition techniques. Firstly, the fabrication of flexible electronics was built on special silicon wafer that allows the silicon nanomembranes (Si NMs) to be released and then transferred to NFC substrate with an adhesive layer, and the device was completed by photoresist patterning and dry etching steps as illustrated in Fig. 4 [271].

**Fig. 5:**
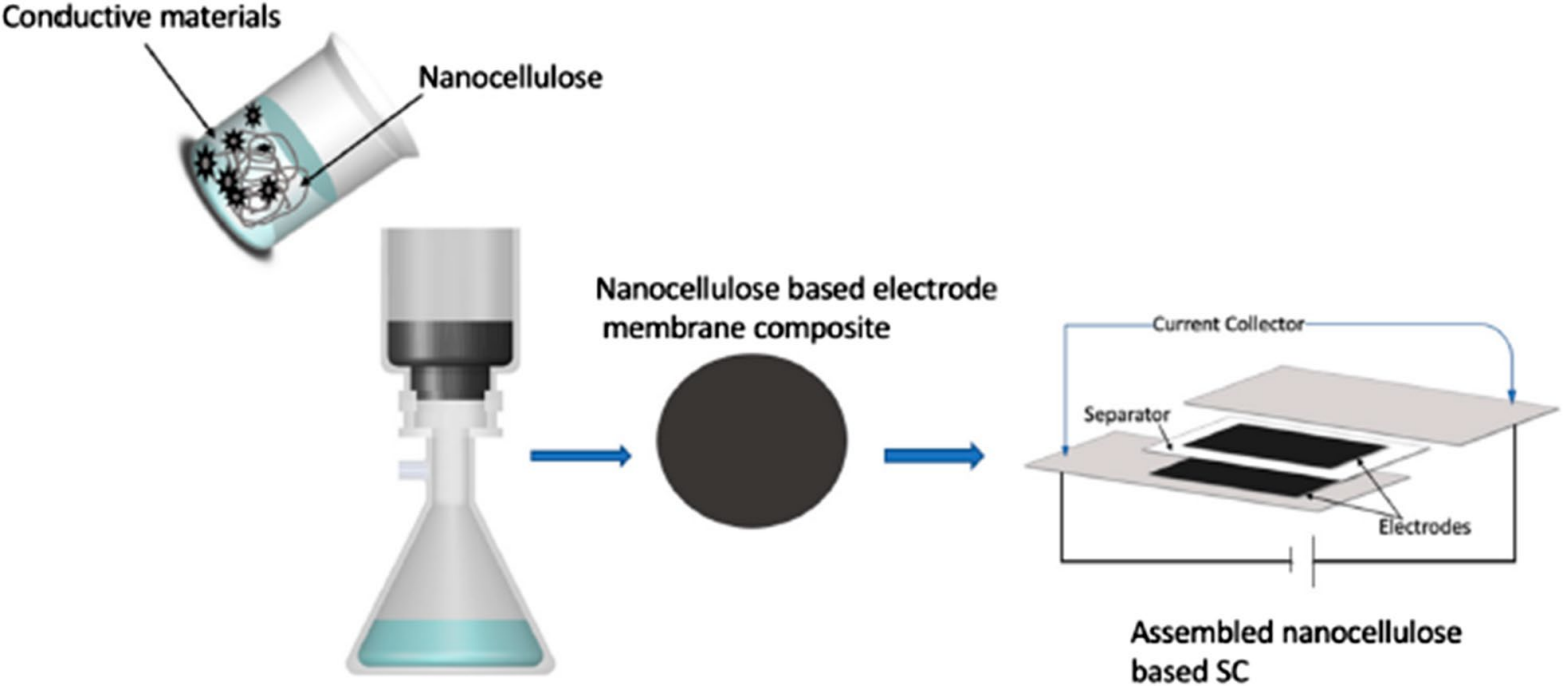
Filtration procedure to fabricate nanocellulose-based composite membrane [supercapacitor (SC)]. *Source*: Hsu and Zhong [244]

Moreover, NFC also is the main substrates for organic light-emitting diodes (OLEDs) (Fig. 5). Okahisa et al. [272] reported that the OLEDs device was fabricated on wood-based nano fibrillated cellulose (NFC) composite.

### Potential of Nanocellulose-Based Composite for Development of Sensor

Nanocellulose has been widely used to develop novel sensors and improve the sensitivity of sensors. In food industry for example, sensors have become an important tool to protect humans from health hazards and risks caused by the food contaminants. Sensors can help to quickly identify mycotoxins, pathogens, heavy metals, pesticides, metal ions, and so on in food. In addition, sensor technology can overcome the complicated, laborious, and time consuming process using expensive instrument that usually requires well-trained personnel [273, 274]. Sensor also provides rapid and sensitive food safety detection.

In the last decade, plenty of sensors (electrochemical sensors, biosensors and chemical sensors) have been successfully developed as alternative or as complementary detection tools for the rapid and sensitive detection [275–277]. However, conventional sensors were developed using plastics, petrochemical-based products and inorganic material which lead to environmental problems. Moreover, issues such as green gas emission, toxicity and sustainability of the materials are becoming increasing. Therefore, the demand for sustainable sensor devices has increased rapidly in recent years.

Nanocelluloses from plants and bacteria have shown promising potentials due to their excellent physical, thermal, mechanical, optical and physical properties, which are important for fabricating high-performance sensor devices. These properties make nanocellulose more preferred as biomaterials since it can enhance the selectivity and sensitivity of sensors for the detection of analytes. On the other hand, the adhesion properties of nanocellulose prevents the leaching problems of immobilised reagents. Thus a remarkable improvement can be obtained for the long-time stability of the sensor. Besides, the hydroxyl –OH groups on nanocellulose can be modified for the incorporation of binding sites for the selective adsorption of different analyte species [278]. The combination of those characteristics makes the nanostructured nanocellulose fibres an ideal building block for conjugation with other functional materials [248, 279, 280] (Table 6).

**Table 6:** Recent biosensor, chemical sensor and electrochemical sensors developed using nanocellulose

Substrate	Reagent/active agent	Application	References
Bacterial nanocellulose	Curcumin	As an albumin assay kit	[281]
Cotton cellulose nanocrystals	Peptide	Detection of human neutropil elastase and a wide range of inlammatory disease	[282]
Polyaniline/crystalline nanocellulose/ionic modified screen-printed electrode	Cholesterol oxidase	As a novel and sensitive electrochemical cholesterol biosensor	[283]
Cellulose nanocrystals	Quartz crystal microbalance	Humidity sensor	[284]
Microfibrillated cellulose/polyvinyl alcohol	Nitrogen modified carbon quantum dot	Tartrazine sensor	[285]

#### Development of Biosensors

**Fig. 6:**
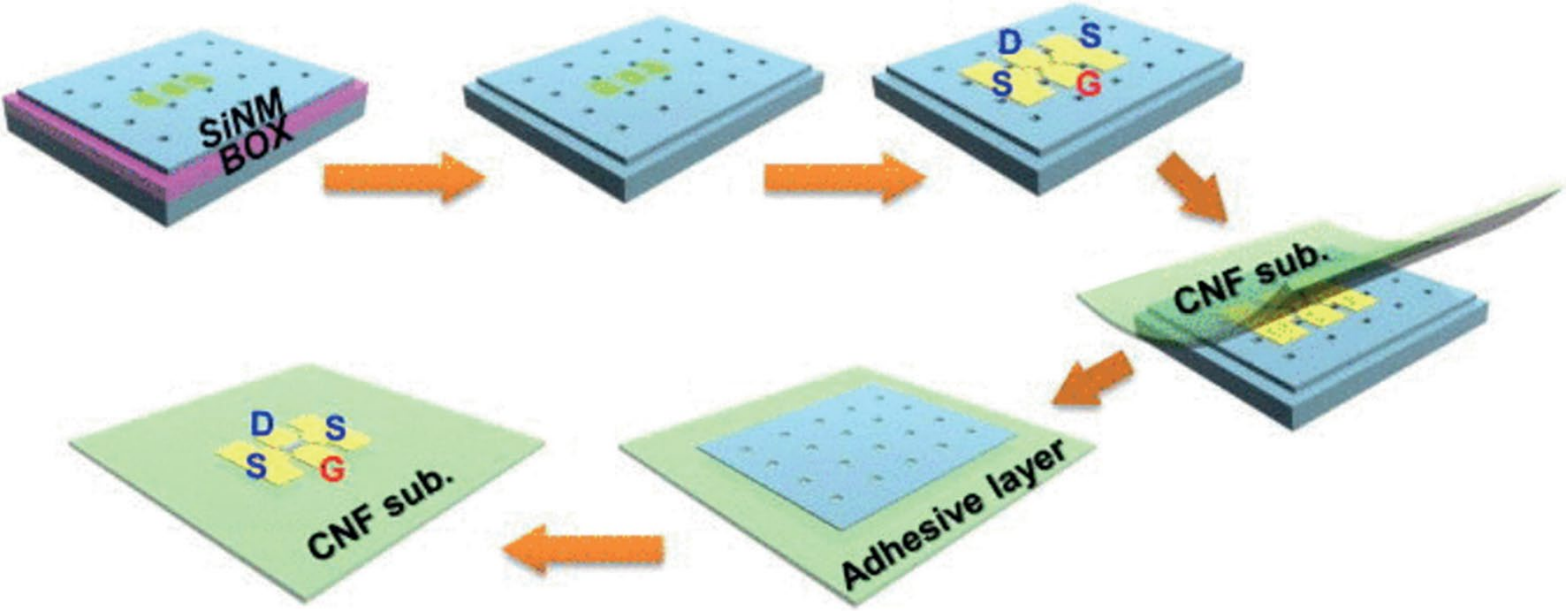
Flexible electronics production process on NFC substrate. *Source*: Sabo et al. [271]

Kim et al. [286] immobilised enzymes candida rigosa lipase into the different cellulose nanocrystals. The findings indicate candida rigisa lipase absorbed on nanocellulose was relatively higher compared to that of microcellulose. This can be related to the high surface area on nanocellulose and thus increase the ionic interaction between nanocellulose anionic group and candida rigisa lipase. In this study, they found that the half-life of the candida rigisa lipase immobilised in nanocellulose increased 27 times higher compared to in free form. Besides, the stability of enzyme also increased (Fig. 6).

**Fig. 7:**
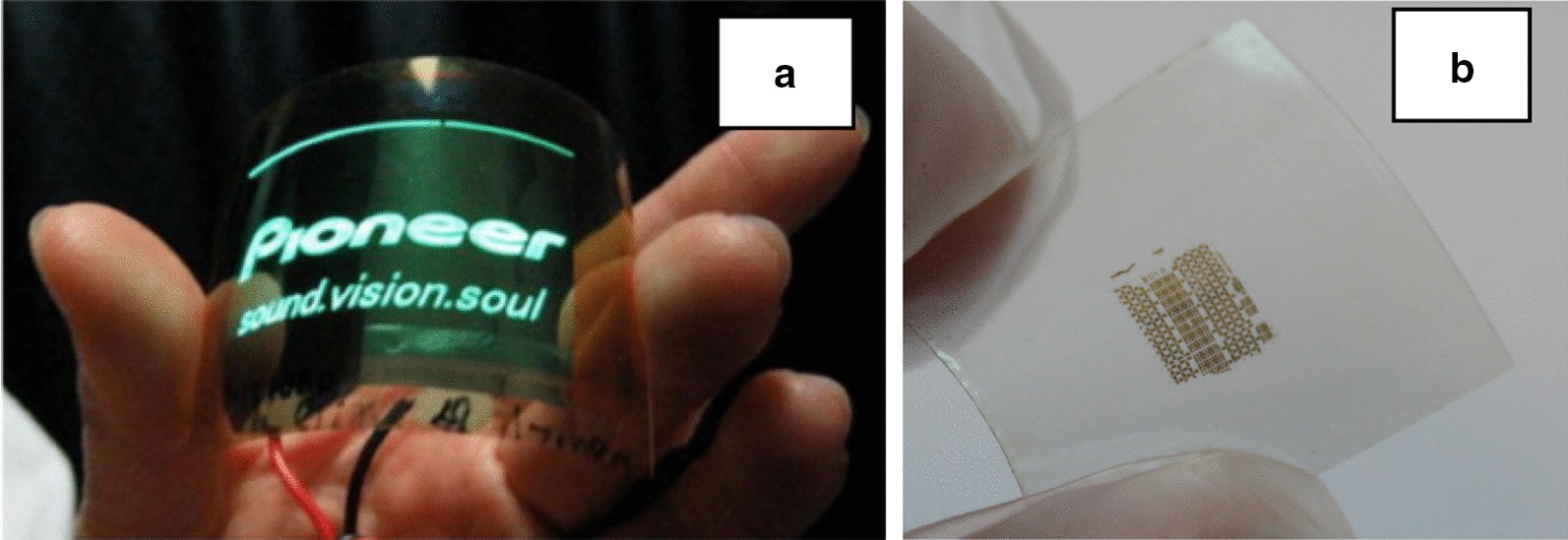
Flexible display (OLEDs) on NFC substrate, **a** *Source*: Okahisa et al. [272]. Reproduced with permission of Elsevier. Flexible electronics on NFC substrate, **b** *Source*: Sabo et al. [271]

Another group of researchers, Edwards et al. [287] studied the kinetic profiles of tri- and tetrapeptides substrate of elastase for the fabrication of elastase biosensor whereby known as human neutrophil elastase (HNE) and porcine pancreatic elastase (PPE). To develop HNE and PPE, immobilised tri- and tetrapeptides were used into cotton cellulose nanocrystals. They found that 2 mg of tripeptide conjugated cotton cellulose nanocrystals in 1 h was able to detect 0.03 U mL^−1^ PPE, while 0.2 mL tetrapeptide conjugated cotton cellulose nanocrystals over 15 min could detect 0.05 U mL^−1^ HNE activity. Incani et al. [288] fabricated a biosensor by immobilising glucose oxidase (GOx) enzyme in a nanocellulose/polyethyleneimine (PEI)/gold nanparticles (AuNPs) nanocomposites. The AuNPs were adsorbed on the cationic PEI and nanocellulose. The Fourier Transform Infrared (FTIR) spectra confirmed that GOx was successfully immobilised on the polymer composites (Fig. 7).

**Fig. 8:**
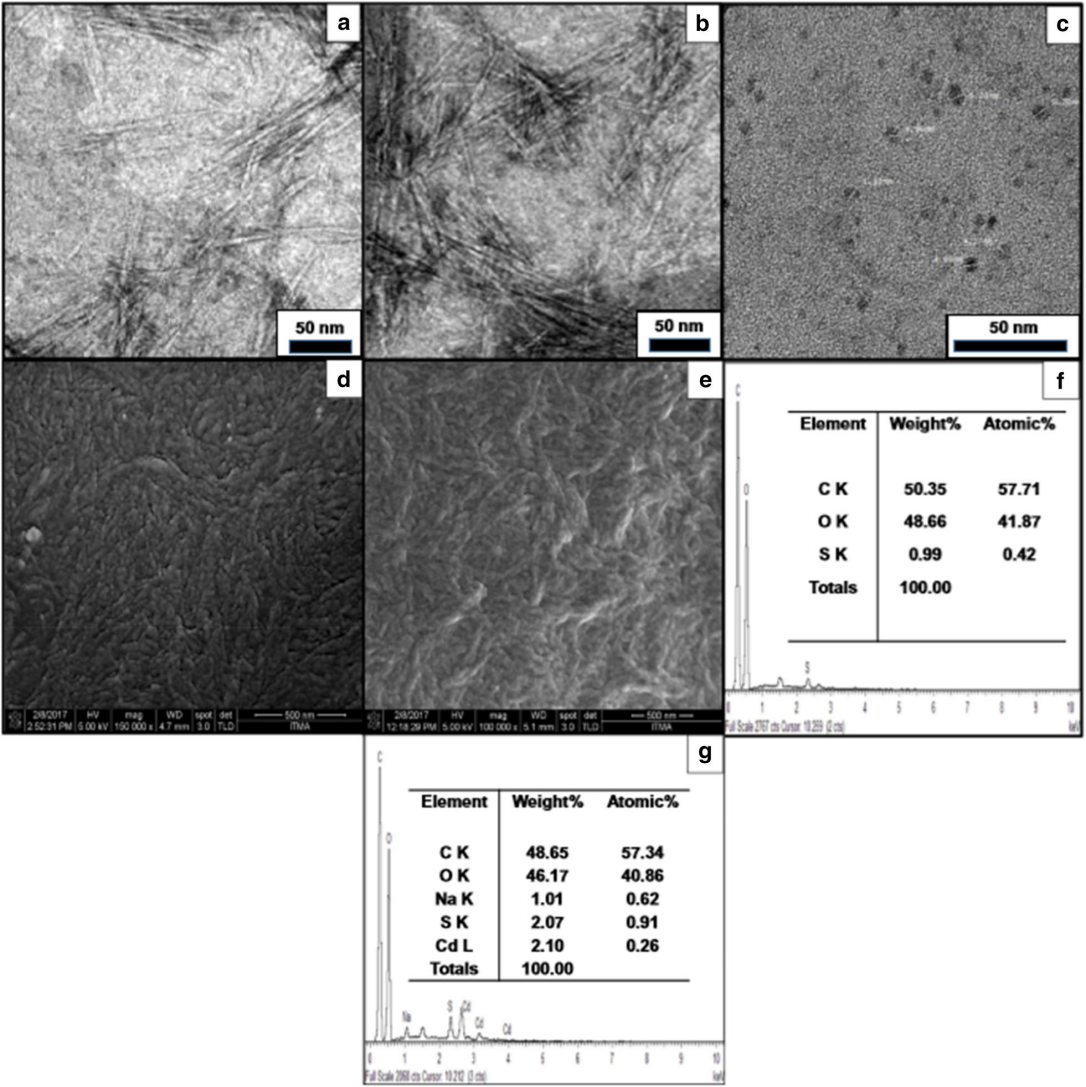
The TEM images for **a** NCC, **b** CTAB-NCC, **c** MPA-QDs, the FESEM micrograph of **d** CTAB-NCC nanostructures film, **e** CTAB-NCC/QDs nanocomposites film, **f** EDX analysis of CTAB-NCC nanostructured film, **g** CTAB-NCC/QDs nanocomposite film (*Source*: Abd Manan et al. [289]). Reproduced with permission of Elsevier

Abd Manan et al. [289] successfully developed biosensor based on nanocrystalline cellulose (NCC)/cadmium sulphide (CdS) quantum dots (QDs) nanocomposites for phenol determination. They modified the NCC with cationic surfactant of cetyltrimethylammonium bromide (CTAB) and further decorated with 3-mercaptopropionic acid (MPA) capped CdS QDs as a scaffold for immobilisation of tyrosinase enzyme (Tyr). The TEM images of NCC and CTAB-NCC (Fig. 8a, b) indicates that agglomerated whiskers-like structure of NCC was not affected by modification, while the MPA QDs are of spherical shape (Fig. 8c). The FESEM images of CTAB-NCC nanostructured film (Fig. 8d) exhibited a homogenous, uniform and dense fibrous structures aggregated, while for CTAB-NCC/QDs nanocomposites film (Fig. 8e), the CdS QDs were appeared as like tiny white dots. EDX analysis shows the presence of respective elemental of carbon (C), oxygen (O), sulphur (S) and cadmium (Cd), indicating the CdS QDs was successfully attached into the CTAB-NCC film. The test finding against phenol shows the biosensor exhibits good linearity towards phenol in the concentration range of 5–40 µM (R2 = 0.9904) with sensitivity and limit of detection of 0.078 µA/µM and 0.082 µM, respectively.

In the food industry, sensors are used to monitor the quality or freshness of food. Moradi et al. [290] developed a pH sensing indicator based on bacterial cellulose nanofibres for monitoring freshness of fish. They used carrot anthocyanins as an indicator. The fabricated sensor displayed wide colour differences from red to gray over the 2–11 pH range. The colour changes were distinguishable whereby deep carmine colour indicates fresh fish, charm pink colour indicates fish is best to eat immediately, and spoiled fish is indicated by jelly bean blue and khaki colours.

#### Development of Chemical Sensors

Nanocellulose hydrogels structure was previously used to immobilise sulphur and nitrogen co-doped graphene quantum dots as a low-cost sensor for detecting the laccase enzyme [291]. Laccase is the multicopper oxidase family of enzyme. It is involved in the monoelectronic oxidation of several aromatic compounds and aliphatic amines. Therefore, it is usually used in decolouration and coloration products [292, 293]. Therefore, monitoring of laccase activity in commercial products is of great interest. From this study, the authors found the sulphur and nitrogen co-doped graphene quantum dots were self-organised via electrostatic interactions.

Faham et al. [294] described the development of a nanocellulose-based colorimetric assay kit for smartphone sensing of iron, Fe(II) and iron-chelating deferoxamine drug (DFO) in biofluids. They embed curcumin in a transparent bacterial cellulose nanopaper, as a colorimetric assay kit. The assay kit was then used for monitoring of iron and deferoxamine as iron-chelating drug in biological fluid such as urine, blood, saliva and serum. The assay kit can be easily coupled with smartphone technology for colorimetric monitoring of Fe(II) and DFO.

#### Development of Electrochemical Sensors

Burrs et al. [295] demonstrated the electrochemical biosensor-based glucose for the detection of pathogenic bacteria. The conductive paper used for the development of electrochemical sensor was prepared using graphene-nanocellulose composites. Platinum was electrodeposited on graphene-cellulose composites using pulsed sonoelectrodeposition [296]. Then the sensor was fabricated by functionalising the nanoplatinum with glucose oxidase (GO_*x*_) entrapped in a chitosan hydrogel on the conductive paper. They found that the sensor is extremely efficient for use in electrochemical biosensing with a low detection limit for glucose or pathogenic bacteria.

Ortolani et al. [297] developed an electrochemical sensing device using nanocellulose and single-walled carbon nanohorns (SWCNH) for guanine and adenine determination. Both nanocellulose and SWCNH have large surface area, good conductivity, high porosity and chemical stability. The prepared sensor showed highly sensitive and high electrocatalytic activity towards simultaneous determination of guanine and adenine. The sensor also presented a lower limit detection. Another study conducted by Shalauddin et al. [298] used hybrid nanocellulose/functionalised multi-walled carbon nanotubes (*f*-MWCNTs for development of electrochemical sensing. The sensor for determination of diclofenac sodium in pharmaceutical drugs and biological fluids samples were used. The presence of –OH groups in the nanocellulose was reported to provide more binding sites for different analytes which ensures an axial modulus rearrangement and incorporation of *f*-MWCNTs.

## Toxicity of Nanomaterial

Although nanomaterial offers many good potential to various industrial sectors, its potential hazard to human and environment could not simply be overlooked [299]. The safety or toxicity issue needs to be addressed by conducting a complete study from various angles. Nanotoxicity or the study of nanomaterials’ toxicity can be classified into several areas namely oxidative stress, genotoxicity, and ecotoxicity. In humans, toxicity can occur through pulmonary, oral and dermal routes. Nanomaterials can affect humans mainly during extraction or production, handling, usage and disposal. Inorganic nanoparticles (NPs) such as ZnO and nickel oxide (NiO) are believed to be more toxic compared to organic nano materials such as nanocellulose. Digested organic NPs are less likely to induce toxicity since the by-products of digestion by stomach acid are simple sugars, although in the case of nanocellulose, the lack of enzyme in human gut capable of degrading cellulose results in the cellulose moving through the gut fairly quickly. A 90-day subchronic toxicity study on Sprague Dawley rats conducted by Ong et al. [300] indicated that no systemic toxicity attributable to 4% dietary consumption of fibrillated cellulose was observed. In the same study, a higher concentration of 30% of nanofibrillated cellulose for 72 weeks also indicated no adverse effects. When present in the air, nanoparticles can also have harmful effects to human health [301]. Multi-walled carbon nanotubes (MWCNT) are possibly one of the most studied NPs for toxicity effects. In the study, Poulsen et al. [302] found that both thick and long as well as thin and short MWCNT induced genotoxic potential and long lasting inflammation and can lead to cardiovascular disease. In contrast, a study on inhalation NCC toxicity in albino rats was conducted and showed no undesirable effects were observed at a maximum concentration of 0.26 mg/L for a period of four (4) hours [303]. At cellular level, cytotoxicity on NCC on different cell types has been studied with most studies concluded that NCC to be non-toxic to a variety of mammalian cells at concentrations below 0.25 mg/L, but can exhibit cytotoxic effects at concentrations of 0.5 mg/L and above [304–309]. Primary nanogenotoxicity refers to the possibility of nanoparticles or their by-products to damage DNA directly or indirectly. Secondary genotoxicity is caused by immune system interaction to disturbances cause by nanoparticles. A study by Akerlund et al. [310] indicated that inorganic NPs of Ni and NiO-induced cytokines inflammations which led to secondary genotoxicity in human lung epithelial cells. However, they require further investigation to understand the factors causing this secondary genotoxicity and whether such results are caused by other nanoparticles as well.

NanoTiO_2_ in wood coatings has been reported to have low toxicity to terrestrial organisms, and no bioaccumulation was observed [311]. Another study conducted on cerium oxide (CeO_2_) nanoparticle or ceria showed that no acute toxicity was observed for Daphnia magna and Thamnocephalus platyurus and D. rerio embryos at test concentrations of 1000, 5000, and 200 mg/L, respectively. However, the same study indicated that a significant chronic toxicity on P. subcapitata was observed with 10% effect concentrations (EC10s) between 2.6 and 5.4 mg/L [312]. Pulido-Reyes et al. [313] explained that the redox cycling between Ce^3+^ and Ce^4+^ is a unique chemistry of nanoceria which depends on the prevailing environmental conditions. They concluded that in most cerium oxide NP studies, the amount of surface Ce^3+^ correlates with toxicity and this can be overcome by blocking the Ce^3+^ sites with anions such as phosphates (PO_4_^3−^) which will reduce reactive oxygen species levels and overcoming oxidative stress. During and after disposal, there is a likelihood that NPs can interact with the environment, whether in soil or aquatic environment. The oxidative stress or carbon-based nanoparticle-induced toxicity is primarily of concern as this condition will affect the oxygen balance in the environment. Wang et al. [314] showed that NCCs’ activated oxidative stress in aquatic organisms (Scenedesmus obliquus algae, planktonic crustacean Daphnia magna and freshwater fish larva Danio rerio) at concentrations above 0.1 mg/L. The NCC form, morphology and concentration are believed to be the contributing factors, thus suggesting evaluation of nanocellulose ecotoxicological impacts when used at large scales. One method of reducing the undesired effect to the environment is by providing a mechanism to capture, retain or recycle the NPs during processing. Magnetic NPs (e.g. magnetite) [315] or surface modification can be used for this purpose. Almost all toxicity studies on nanoparticles recommend more research to be conducted on finding suitable analytical techniques and protocols in detecting organic NPs within biological matrices as well as mechanistic and environmental investigations to understand potential NPs risks on the ecosystem [301, 302, 310, 316].

## Conclusions

The potential of nanotechnology in wood-based products sector is enormous. Nanotechnology offers the opportunity to change the landscape of wood-based products industry locally and globally. Forest itself via forest plantation can provide sustainable sources towards the creation of new generation of cellulose called nanocellulose that could offer myriads of application in various industrial fields. Besides, nanocellulose is less expensive than carbon-based material (e.g. carbon nanotube, graphene), environmentally friendly and may improve the recyclability and performance of countless products. For instance, nanocellulose can be used to improve the performance of paper and reduce the use of pulp in papermaking, thus resulting in cost saving. Nanocellulose can also be made into nanopaper or thin film for use in paper packaging or other sector such as electronics which offers alternative to non-renewable plastic. Nanocellulose used in wood composite can lead to the development of advanced composites which can be tailored to specific uses. Together with their strength properties and affordability makes them a viable solution to reduce the need for solid wood. Industrial sectors other than wood-based products could also benefit from the use of nanocellulose such as in energy device and sensors. Various nanocellulose-based products for energy storage and energy harvester applications as well as sensor have been developed at laboratory scale. In view of this interesting development, Malaysia has embarked on the production of nanocellulose from various tropical lignocellulosic materials. Unfortunately, the application of nanocellulose at commercial scale in Malaysia is still non-existent. Thus, it requires collective, concert and intensive efforts from various players that include stakeholders, industry and researchers to promote the use of nanocellulose and ensure that nanocellulose could enter the next level of application and commercialisation stage. The use of nanosized material other than nanocellulose has already benefited and also given advantage to wood-based products area which includes pulp and paper, wood composite, wood coating and wood preservative.

## Data Availability

All data and materials are available without restriction.

## Abbreviations

AgNPs: Silver nanoparticles
Al_2_O_3_: Aluminium oxide
AuNPs: Gold nanparticles
BTZ: Benzotriazole
C: Carbon
CCA: Chromated copper arsenate
Cd: Cadmium
CdS: Cadmium sulphide
CeO_2_: Cerium oxide
CH_2_CHSi(OC_2_H_5_)_3_: Vinyltriethoxysilane
CMC: Carboxymethyl cellulose
CNT: Carbon nanotubes
CO_2_: Carbon dioxide
CTAB: Cetyltrimethylammonium bromide
CTAB-NCC: Cetyltrimethylammonium bromide-nanocrystalline cellulose
CWA: Castor-oil-based waterborne acrylate (CWA)
DFO: Deferoxamine drug
FESEM: Field-emission scanning electron microscopy
*f*-MWCNTs: Functionalised multi-walled carbon nanotubes
FTIR: Fourier transform Infrared
GOx: Glucose oxidase
HALS: Hindered amine light stabilizers
HNE: Human neutrophil elastase
ISE: Inorganic solid electrolyte
LDH: Layered double hydroxide
LIBs: Lithium-ion battery
LiTFSI: Bis(trifluoromethanesulphonyl)imide lithium salt
L-NCC: Lignin-coated nanocrystalline cellulose (L-NCC)
MC: Moisture content
Mg–Al LDH: Magnesium–aluminium layered double hydroxide
MPA: 3-Mercaptopropionic acid
MUF: Melamine-urea formaldehyde
MWCNT: Multi-walled carbon nanotube
NCC: Nanocrystalline cellulose
NFC: Nanofibrillated cellulose
Ni: Nickel
NiO: Nickel oxide
NPs: Nanoparticles
NW: Nanowollastonite
O: Oxygen
OH: Hydroxyl
OLEDs: Organic light-emitting diodes
PA: Polyamide
PCL: Polycaprolactone
PCP: Pentachlorophenol
PDMS: Polydimethylsiloxane
PE: Polyethylene
PEG: Poly(ethylene glycol)
PEO: Polyethylene oxide
PHB: Poly(3-hydroxybutyrate-co-4-hydroxybutyrate)
PHRR: Peak heat release rate
PLA: Polylactic acid
PMC: Perfluoroalkyl methacrylic copolymer (PMC)
PMMA: Poly(methyl methacrylate)
PO_4_^3−^: Phosphates
POTS: Perfluoroalkyltriethoxysilane
PP: Polypropylene
PPE: Porcine pancreatic elastase
PPG: Propylene glycol
PS: Polystyrene
PSU: Polysulphone
PU: Polyurethane
PV: Photovoltaic
QDs: Quantum dots
RFID: Radio-frequency identification
S: Sulphur
SC: Supercapacitor
Si NMs: Silicon nanomembranes
SiO_2_: Silicon oxide
SPE: Solid polymer electrolyte
SPI: Soy protein isolate
SWCNH: Single-walled carbon nanohorns
TBOT: Tetrabutyltitanate
TEM: Transmission electron microscopy
TEMPO: 2,2,6,6-Tetramethylpiperidin-1-oxyl
Ti(OC4H9)4: Tetrabutyltitanate
TiO_2_: Titanium dioxide
TMI-BNT: Transition metal ion-modified bentonite
TNFC: TEMPO-oxidised nanofibrillated cellulose
Tyr: Tyrosinase
UF: Urea formaldehyde
UP: Unsaturated polyester
UV: Ultraviolet
VRFBs: Vanadium redox flow batteries
VTES: Vinyltriethoxysilane
WPU: Waterborne polyurethane coating
WUV: Waterborne
Zn-Al LDH: Zinc-aluminium layered double hydroxide
ZnO: Zinc oxide
ZnSt_2_: Zinc stearate
ZnSt_2_/WUV: Zinc stearate/waterborne UV

## References

[1] Wegner TH, Jones EP (2009). A fundamental review of the relationships between nanotechnology and lignocellulosic biomass. Nanosci Technol Renew Biomater.

[2] Wegner TH, Jones PE (2006). Advancing cellulose-based nanotechnology. Cellulose.

[3] Bajpai P (2016). Pulp and paper industry: nanotechnology in forest industry.

[4] Malaysia Timber Industry Board (MTIB). Malaysian timber E-stats (2019)

[5] Moon RJ, Martini A, Nairn J, Simonsen J, Youngblood J (2011). Cellulose nanomaterials review: structure, properties and nanocomposites. Chem Soc Rev.

[6] Klemm D, Kramer F, Moritz S, Lindström T, Ankerfors M, Gray D (2011). Nanocelluloses: a new family of nature-based materials. Angew Chemie Int Ed (Internet).

[7] Köse K, Mavlan M, Youngblood JP (2020). Applications and impact of nanocellulose based adsorbents. Cellulose.

[8] Trache D, Tarchoun AF, Derradji M, Hamidon TS, Masruchin N, Brosse N (2020). Nanocellulose: from fundamentals to advanced applications. Front Chem.

[9] Phanthong P, Reubroycharoen P, Hao X, Xu G, Abudula A, Guan G (2018). Nanocellulose: extraction and application. Carbon Resour Convers.

[10] Shatkin JA, Wegner TH, Bilek EMT, Cowie J (2014). Market projections of cellulose nanomaterial-enabled products—part 1: applications. Tappi J.

[11] Cowie J, Bilek EMT, Wegner TH, Shatkin JA (2014). Market projections of cellulose nanomaterial-enabled products—part 2: volume estimates. Tappi J.

[12] Klemm D, Cranston ED, Fischer D, Gama M, Kedzior SA, Kralisch D (2018). Nanocellulose as a natural source for groundbreaking applications in materials science: today’s state. Mater Today.

[13] Thomas B, Raj MC, Joy J, Moores A, Drisko GL, Sanchez C (2018). Nanocellulose, a versatile green platform: from biosources to materials and their applications. Chem Rev.

[14] De Filpo G, Palermo AM, Rachiele F, Nicoletta FP (2013). Preventing fungal growth in wood by titanium dioxide nanoparticles. Int Biodeterior Biodegrad.

[15] Cristea MV, Riedl B, Blanchet P (2011). Effect of addition of nanosized UV absorbers on the physico-mechanical and thermal properties of an exterior waterborne stain for wood. Prog Org Coat.

[16] Francés Bueno AB, Bañón N, Martínez de Morentín L, Moratalla García J (2014). Treatment of natural wood veneers with nano-oxides to improve their fire behaviour. MS&E.

[17] Salma U, Chen N, Richter DL, Filson PB, Dawson-Andoh B, Matuana L (2010). Amphiphilic core/shell nanoparticles to reduce biocide leaching from treated wood, 1-leaching and biological efficacy. Macromol Mater Eng.

[18] Liu Y, Laks P, Heiden P (2002). Controlled release of biocides in solid wood. I. Efficacy against brown rot wood decay fungus (*Gloeophyllum trabeum*). J Appl Polym Sci.

[19] Peteu SF, Oancea F, Sicuia OA, Constantinescu F, Dinu S (2010). Responsive polymers for crop protection. Polymers (Basel).

[20] Moon RJ, Frihart CR, Wegner T (2006). Nanotechnology applications in the forest products industry. For Prod J.

[21] McCrank J (2009). Nanotechnology applications in the forest sector.

[22] Julkapli NM, Bagheri S (2016). Developments in nano-additives for paper industry. J Wood Sci.

[23] Statista (2020) Global paper production volume from 2007 to 2017 by type

[24] Taipale T, Osterberg M, Nykanen A, Ruokolainen J, Laine J (2010). Effect of microfibrillated cellulose and fines on the drainage of kraft pulp suspension and paper strength. Cellulose.

[25] Boufi S (2016). Nanofibrillated cellulose as an additive in papermaking process: a review. Carbohydr Polym.

[26] Jasmani L, Adnan S (2017). Preparation and characterization of nanocrystalline cellulose from Acacia mangium and its reinforcement potential. Carbohydr Polym.

[27] Zhao J, Zhang W, Zhang X, Zhang X, Lu C, Deng Y (2013). Extraction of cellulose nanofibrils from dry softwood pulp using high shear homogenization. Carbohydr Polym.

[28] Latifah J, Nurrul-Atika M, Sharmiza A, Rushdan I (2020). Extraction of nanofibrillated cellulose from Kelempayan (Neolamarckia cadamba) and its use as strength additive in papermaking. J Trop For Sci.

[29] Beck-Candanedo S, Roman M, Gray DG (2005). Effect of reaction conditions on the properties and behavior of wood cellulose nanocrystal suspensions. Biomacromol.

[30] Jonoobi M, Harun J, Mathew AP, Oksman K (2010). Mechanical properties of cellulose nanofiber (CNF) reinforced polylactic acid (PLA) prepared by twin screw extrusion. Compos Sci Technol.

[31] Garcia de Rodriguez NL, Thielemans W, Dufresne A (2006). Sisal cellulose whiskers reinforced polyvinyl acetate nanocomposites. Cellulose.

[32] Malainine ME, Mahrouz M, Dufresne A (2005). Thermoplastic nanocomposites based on cellulose microfibrils from Opuntia ficus-indica parenchyma cell. Compos Sci Technol.

[33] Leitner J, Hinterstoisser B, Wastyn M, Keckes J, Gindl W (2007). Sugar beet cellulose nanofibril-reinforced composites. Cellulose.

[34] Saito T, Kimura S, Nishiyama Y, Isogai A (2007). Cellulose nanofibres prepared by tempo-mediated oxidation of native cellulose. Biomacromol.

[35] Pääkkö M, Ankerfors M, Kosonen H, Nykänen A, Ahola S, Österberg M (2007). Enzymatic hydrolysis combined with mechanical shearing and high-pressure homogenization for nanoscale cellulose fibrils and strong gels. Biomacromol.

[36] Henriksson M, Henriksson G, Berglund LA, Lindström T (2007). An environmentally friendly method for enzyme-assisted preparation of microfibrillated cellulose (MFC) nanofibers. Eur Polym J.

[37] Abe K, Iwamoto S, Yano H (2007). Obtaining cellulose nanofibers with a uniform width of 15 nm from wood. Biomacromol.

[38] Hult E-L, Larsson PT, Iversen T (2001). Cellulose fibril aggregation—an inherent property of kraft pulps. Polymer (Guildf).

[39] Frone AN, Panaitescu DM, Donescu D (2011). Some aspects concerning the isolation of cellulose micro- and nano-fibers. UPB Sci Bull Ser B Chem Mater Sci.

[40] Bhatnagar A, Sain M (2005). Processing of cellulose nanofiber-reinforced composites. J Reinf Plast Compos.

[41] Chakraborty A, Sain M, Kortschot M (2005). Cellulose microfibrils: a novel method of preparation using high shear refining and cryocrushing. Holzforschung (Internet).

[42] Alemdar A, Sain M (2008). Biocomposites from wheat straw nanofibers: morphology, thermal and mechanical properties. Compos Sci Technol.

[43] Wang B, Sain M (2007). Isolation of nanofibers from soybean source and their reinforcing capability on synthetic polymers. Compos Sci Technol.

[44] Saito T, Kimura S, Nishiyama Y, Isogai A (2007). Cellulose nanofibers prepared by TEMPO-mediated oxidation of native cellulose. Biomacromol.

[45] Man Z, Muhammad N, Sarwono A, Bustam MA, Kumar MV, Rafiq S (2011). Preparation of cellulose nanocrystals using an ionic liquid. J Polym Environ.

[46] Henriksson M, Berglund LA (2007). Structure and properties of cellulose nanocomposite films containing melamine formaldehyde. J Appl Polym Sci.

[47] Siqueira G, Tapin-Lingua S, Bras J, Perez DD, Dufresne A (2020). Morphological investigation of nanoparticles obtained from combined mechanical shearing, and enzymatic and acid hydrolysis of sisal fibers. Cellulose.

[48] Nickerson RF, Habrle JA (1947). Cellulose intercrystalline structure. Study by hydrolytic methods. Ind Eng Chem.

[49] Araki J (2013). Electrostatic or steric? Preparations and characterizations of well-dispersed systems containing rod-like nanowhiskers of crystalline polysaccharides. Soft Matter.

[50] Dong XM, Revol JF, Gray DG (1998). Effect of microcrystallite preparation conditions on the formation of colloid crystals of cellulose. Cellulose.

[51] Araki J, Wada M, Kuga S, Okano T (1998). Flow properties of microcrystalline cellulose suspension prepared by acid treatment of native cellulose. Colloids Surfaces A Physicochem Eng Asp.

[52] Bai W, Holbery J, Li K (2009). A technique for production of nanocrystalline cellulose with a narrow size distribution. Cellulose.

[53] Bondeson D, Mathew A, Oksman K (2006). Optimization of the isolation of nanocrystals from microcrystalline cellulose by acid hydrolysis. Cellulose.

[54] Azizi Samir MAS, Alloin F, Sanchez JY, El Kissi N, Dufresne A (2004). Preparation of cellulose whiskers reinforced nanocomposites from an organic medium suspension. Macromolecules.

[55] Dufresne A (2017). Nanocellulose: from nature to high performance tailored materials.

[56] González I, Boufi S, Pèlach MA, Alcalà M, Vilaseca F, Mutjé P (2012). Nanofibrillated cellulose as paper additive in eucalyptus pulps. BioResources.

[57] Gonzalez I (2014). From paper to nanopaper: evolution of mechanical and physical properties. Cellulose (Internet).

[58] Eriksen O, Syverud K, Gregersen O (2008). The use of microfibrillated cellulose produced from kraft pulp as strength enhancer in TMP paper. Nord Pulp Pap Res J (Internet).

[59] Manninen M, Kajanto I, Happonen J, Paltakari J (2011). The effect of microfibrillated cellulose addition on drying shrinkage and dimensional stability of wood-free paper. Nord Pulp Pap Res J.

[60] Henriksson M, Berglund LA, Isaksson P, Lindström T, Nishino T (2008). Cellulose nanopaper structures of high toughness. Biomacromol.

[61] Balea A (2020). Industrial application of nanocelluloses in papermaking: a review of challenges, technical solutions, and market perspectives. Molecules (Internet).

[62] Ahola S, Österberg M, Laine J (2008). Cellulose nanofibrils—adsorption with poly(amideamine) epichlorohydrin studied by QCM-D and application as a paper strength additive. Cellulose.

[63] Minor JL, Atalia RH, Harten TM (1993). Improving interfibre bonding of recycled fibres. J Pulp Pap Sci.

[64] Hubbe MA (2006). Bonding between cellulosic fibers in the absence and presence of dry-strength agents—a review. BioResources.

[65] Hii C, Gregersen ØW, Chinga-Carrasco G, Eriksen Ø (2012). The effect of MFC on the pressability and paper properties of TMP and GCC based sheets. Nord Pulp Pap Res J.

[66] Lin T, Yin X, Retulainen E, Nazhad MM (2007). Effect of chemical pulp fines on filler retention and paper properties. Appita Technol Innov Manuf Environ.

[67] Guimond R, Chabot B, Law KN, Daneault C (2010). The use of cellulose nanofibres in papermaking. J Pulp Pap Sci.

[68] Zhang X, Lin Z, Chen B, Sharma S, Wong C, Zhang W (2013). Solid-state, flexible, high strength paper-based supercapacitors. J Mater Chem A.

[69] Lavoine N, Desloges I, Khelifi B, Bras J (2014). Impact of different coating processes of microfibrillated cellulose on the mechanical and barrier properties of paper. J Mater Sci.

[70] Aulin C, Ström G (2013). Multilayered alkyd resin/nanocellulose coatings for use in renewable packaging solutions with a high level of moisture resistance. Ind Eng Chem Res.

[71] Syverud K, Stenius P (2009). Strength and barrier properties of MFC films. Cellulose.

[72] Hult E-L, Iotti M, Lenes M (2010). Efficient approach to high barrier packaging using microfibrillar cellulose and shellac. Cellulose.

[73] Nogi M, Handa K, Nakagaito AN, Yano H (2005). Optically transparent bionanofiber composites with low sensitivity to refractive index of the polymer matrix. Appl Phys Lett.

[74] Fukahori S, Ichiura H, Kitaoka T, Tanaka H (2003). Photocatalytic decomposition of bisphenol a in water using composite TiO_2_–zeolite sheets prepared by a papermaking technique. Environ Sci Technol.

[75] Li B, Li Y, Kang Y, Yang W (2009). Surface modification of nanoTiO_2_ for paper coatings. China Pulp Pap.

[76] Futures Markets Inc. (FMI) (2012) The global market for nanocellulose to 2017

[77] Juuti M, Koivunen K, Silvennoinen M, Paulapuro H, Peiponen K-E (2009). Light scattering study from nanoparticle-coated pigments of paper. Colloids Surf A Physicochem Eng Asp.

[78] Wild MP, Wildlock YM, Andersson KR, Lindgren E (2008) A new paper coating nanotechnology with unique characteristics to improve inkjet print density

[79] Yan A, Liu Z, Miao R (2010). Photocatalytic degradation of xylene by nano-TiO_2_/β-cyclodextrin coated paper. China Pulp Pap.

[80] Atik C, Ates S (2012). Mass balance of silica in straw from the perspective of silica reduction in straw pulp. BioResources.

[81] Wu W, Jing Y, Zhou X, Dai H (2011) Preparation and properties of cellulose fiber/silica core–shell magnetic nanocomposites. In: 16th international symposium on wood, fiber and pulping chemistry—proceedings, ISWFPC, Tiajin, pp 1277–1282

[82] Liu QX, Xu WC, Lv Y Bin, Li JL (2011) Application of precipitated silica in low basis weight newspaper. In: Advanced materials research. Trans Tech Publ, New York, pp 1107–1111

[83] Gan PG, Sam ST, Abdullah MFB, Omar MF (2020). Thermal properties of nanocellulose-reinforced composites: a review. J Appl Polym Sci.

[84] Mondal S (2018). Review on nanocellulose polymer nanocomposites. Polym Plast Technol Eng.

[85] Fujisawa S, Togawa E, Kimura S (2018). Large specific surface area and rigid network of nanocellulose govern the thermal stability of polymers: mechanisms of enhanced thermomechanical properties for nanocellulose/PMMA nanocomposite. Mater Today Commun.

[86] Geng S, Haque MM-U, Oksman K (2016). Crosslinked poly(vinyl acetate) (PVAc) reinforced with cellulose nanocrystals (CNC): structure and mechanical properties. Compos Sci Technol.

[87] Pinheiro IF, Ferreira FV, Souza DHS, Gouveia RF, Lona LMF, Morales AR (2017). Mechanical, rheological and degradation properties of PBAT nanocomposites reinforced by functionalized cellulose nanocrystals. Eur Polym J.

[88] Fan J, Njuguna J (2016) An introduction to lightweight composite materials and their use in transport structures. In: Lightweight composite structures in transport. Elsevier, Berlin, pp 3–34

[89] Trappe V, Günzel S, Jaunich M (2012). Correlation between crack propagation rate and cure process of epoxy resins. Polym Test.

[90] Lanna A, Suklueng M, Kasagepongsan C, Suchat S (2020). Performance of novel engineered materials from epoxy resin with modified epoxidized natural rubber and nanocellulose or nanosilica. Adv Polym Technol.

[91] Saba N, Jawaid M, Alothman OY, Almutairi Z (2019). Evaluation of dynamic properties of nano oil palm empty fruit bunch filler/epoxy composites. J Mater Res Technol.

[92] Nissilä T, Hietala M, Oksman K (2019). A method for preparing epoxy-cellulose nanofiber composites with an oriented structure. Compos Part A Appl Sci Manuf.

[93] Ansari F, Skrifvars M, Berglund L (2015). Nanostructured biocomposites based on unsaturated polyester resin and a cellulose nanofiber network. Compos Sci Technol.

[94] Gan L, Liao J, Lin N, Hu C, Wang H, Huang J (2017). Focus on gradientwise control of the surface acetylation of cellulose nanocrystals to optimize mechanical reinforcement for hydrophobic polyester-based nanocomposites. ACS Omega.

[95] Niu X, Liu Y, Song Y, Han J, Pan H (2018). Rosin modified cellulose nanofiber as a reinforcing and co-antimicrobial agents in polylactic acid/chitosan composite film for food packaging. Carbohydr Polym.

[96] Geng S, Wei J, Aitomäki Y, Noël M, Oksman K (2018). Well-dispersed cellulose nanocrystals in hydrophobic polymers by in situ polymerization for synthesizing highly reinforced bio-nanocomposites. Nanoscale.

[97] Spagnol C, Fragal EH, Witt MA, Follmann HDM, Silva R, Rubira AF (2018). Mechanically improved polyvinyl alcohol-composite films using modified cellulose nanowhiskers as nano-reinforcement. Carbohydr Polym.

[98] O’Donnell KL, Oporto-Velásquez GS, Comolli N (2020). Evaluation of acetaminophen release from biodegradable poly(vinyl alcohol) (PVA) and nanocellulose films using a multiphase release mechanism. Nanomaterials.

[99] Alves JS, Dos Reis KC, Menezes EGT, Pereira FV, Pereira J (2015). Effect of cellulose nanocrystals and gelatin in corn starch plasticized films. Carbohydr Polym.

[100] Larraza I, Vadillo J, Santamaria-Echart A, Tejado A, Azpeitia M, Vesga E (2020). The effect of the carboxylation degree on cellulose nanofibers and waterborne polyurethane/cellulose nanofiber nanocomposites properties. Polym Degrad Stab.

[101] Wang L, Okada K, Hikima Y, Ohshima M, Sekiguchi T, Yano H (2019). Effect of cellulose nanofiber (CNF) surface treatment on cellular structures and mechanical properties of polypropylene/CNF nanocomposite foams via core-back foam injection molding. Polymers (Basel).

[102] Palacios Hinestroza H, Urena-Saborio H, Zurita F, Guerrero de León AA, Sundaram G, Sulbarán-Rangel B (2020). Nanocellulose and polycaprolactone nanospun composite membranes and their potential for the removal of pollutants from water. Molecules.

[103] Rehim MHA, Yassin MA, Zahran H, Kamel S, Moharam ME, Turky G (2019). Rational design of active packaging films based on polyaniline-coated polymethyl methacrylate/nanocellulose composites. Polym Bull.

[104] Maia E, Peguy A, Perez S (1981). Cellulose organic solvents. (I): the structure of anhydrous *n*-methylmorpholine *n*-oxide and *n*-methylmorpholine *n*-oxide monohydrate. Acta Crystallogr B.

[105] Onbattuvelli VP, Enneti RK, Simonsen J, Kate KH, Balla VK, Atre SV (2020). Structure and thermal stability of cellulose nanocrystal/polysulfone nanocomposites. Mater Today Commun.

[106] Bao Y, Zhang H, Luan Q, Zheng M, Tang H, Huang F (2018). Fabrication of cellulose nanowhiskers reinforced chitosan-xylan nanocomposite films with antibacterial and antioxidant activities. Carbohydr Polym.

[107] Beuguel Q, Tavares JR, Carreau PJ, Heuzey M-C (2018). Rheological behavior of cellulose nanocrystal suspensions in polyethylene glycol. J Rheol (N Y).

[108] Jeon JG, Kim HC, Kim J, Kang TJ (2020). Polystyrene nanocomposites reinforced with phenyl isocyanate-treated cellulose nanofibers. Funct Compos Struct.

[109] Kashani Rahimi S, Otaigbe JU (2016). Polyamide 6 nanocomposites incorporating cellulose nanocrystals prepared by in situ ring-opening polymerization: viscoelasticity, creep behavior, and melt rheological properties. Polym Eng Sci.

[110] Oun AA, Rhim J-W (2017). Characterization of carboxymethyl cellulose-based nanocomposite films reinforced with oxidized nanocellulose isolated using ammonium persulfate method. Carbohydr Polym.

[111] Hammami I, Benhamou K, Hammami H, SoretoTeixeira S, Arous M, Kaddami H (2020). Electrical, morphology and structural properties of biodegradable nanocomposite polyvinyl-acetate/cellulose nanocrystals. Mater Chem Phys.

[112] Ashori A, Jonoobi M, Ayrilmis N, Shahreki A, Fashapoyeh MA (2019). Preparation and characterization of polyhydroxybutyrate-co-valerate (PHBV) as green composites using nano reinforcements. Int J Biol Macromol.

[113] Ingole VH, Vuherer T, Maver U, Vinchurkar A, Ghule AV, Kokol V (2020). Mechanical properties and cytotoxicity of differently structured nanocellulose-hydroxyapatite based composites for bone regeneration application. Nanomaterials.

[114] Papadopoulos AN (2010). Chemical modification of solid wood and wood raw materials for composites production with linear chain carboxylic acid anhydrides: a brief review. BioResources.

[115] Reinprecht L, Iždinský J, Vidholdová Z (2018). Biological resistance and application properties of particleboards containing nano-zinc oxide. Adv Mater Sci Eng.

[116] Nosal E, Reinprecht L (2019). Anti-bacterial and anti-mold efficiency of silver nanoparticles present in melamine-laminated particleboard surfaces. BioResources.

[117] Gao W, Du G (2015). Physico-mechanical properties of plywood bonded by nano cupric oxide (CuO) modified pf resins against subterranean termites. Maderas Cienc Tecnol.

[118] Abd Norani K, Hashim R, Sulaiman O, Hiziroglu S, Ujang S, Noor W (2017). Biodegradation behaviour of particleboard bonded with modified PVOH/oil palm starch and nano silicon dioxide. Iran J Energy Environ.

[119] Silva LCL, Lima FO, Chahud E, Christoforo AL, Lahr FAR, Favarim HR (2019). Heat transfer and physical-mechanical properties analysis of particleboard produced with ZnO nanoparticles addition. BioResources.

[120] Gupta A, Kumar A, Sharma KV, Gupta R (2018). Application of high conductive nanoparticles to enhance the thermal and mechanical properties of wood composite. Mater Today Proc.

[121] Muñoz F, Moya R (2018). Effect of nanoclay-treated UF resin on the physical and mechanical properties of plywood manufactured with wood from tropical fast growth plantations. Maderas Cienc Tecnol.

[122] Wang X, Wang S, Xie X, Zhao L, Deng Y, Li Y (2017). Multi-scale evaluation of the effects of nanoclay on the mechanical properties of wood/phenol formaldehyde bondlines. Int J Adhes Adhes.

[123] Wibowo ES, Lubis MAR, Park B-D, Kim JS, Causin V (2020). Converting crystalline thermosetting urea–formaldehyde resins to amorphous polymer using modified nanoclay. J Ind Eng Chem.

[124] Taghiyari HR, Nouri P (2015). Effects of nano-wollastonite on physical and mechanical properties of medium-density fiberboard. Maderas Cienc Tecnol.

[125] Taghiyari HR, Commons LC (2018). Effects of adding nano-wollastonite, date palm prunings and two types of resins on the physical and mechanical properties of medium-density fibreboard (MDF) made from wood fibres. Bois Forêts des Trop.

[126] da Silva APS, Ferreira BS, Favarim HR, Silva MFF, Silva JVF, dos Anjos AM (2019). Physical properties of medium density fiberboard produced with the addition of ZnO nanoparticles. BioResources.

[127] Candan Z, Akbulut T (2015). Physical and mechanical properties of nanoreinforced particleboard composites. Maderas Cienc Tecnol.

[128] Taghiyari HR, Norton J (2014). Effect of silver nanoparticles on hardness in medium-density fiberboard (MDF). iForest Biogeosci For.

[129] Kawalerczyk J, Dziurka D, Mirski R, Szentner K (2020). Properties of plywood produced with urea-formaldehyde adhesive modified with nanocellulose and microcellulose. Drv Ind Znan Časopis za Pitanja Drv Tehnol.

[130] Hansted FAS, Hansted ALS, Padilha ERD, Caraschi JC, Goveia D, de Campos CI (2019). The use of nanocellulose in the production of medium density particleboard panels and the modification of its physical properties. BioResources.

[131] Amini E, Tajvidi M, Gardner DJ, Bousfield DW (2017). Utilization of cellulose nanofibrils as a binder for particleboard manufacture. BioResources.

[132] Hunt JF, Leng W, Tajvidi M (2017). Vertical density profile and internal bond strength of wet-formed particleboard bonded with cellulose nanofibrils. Wood Fiber Sci.

[133] Leng W, Hunt JF, Tajvidi M (2017). Screw and nail withdrawal strength and water soak properties of wet-formed cellulose nanofibrils bonded particleboard. BioResources.

[134] Fengel D, Wegener G (2003). Wood chemistry, ultrastructure, reaction.

[135] Sandberg D (2016) Additives in wood products—today and future development. In: Environmental impacts of traditional and innovative forest-based bioproducts. Springer, London, pp 105–172

[136] Hincapié I, Künniger T, Hischier R, Cervellati D, Nowack B, Som C (2015). Nanoparticles in facade coatings: a survey of industrial experts on functional and environmental benefits and challenges. J Nanoparticle Res.

[137] Okyay TO, Bala RK, Nguyen HN, Atalay R, Bayam Y, Rodrigues DF (2015). Antibacterial properties and mechanisms of toxicity of sonochemically grown ZnO nanorods. RSC Adv.

[138] Chakra C, Raob K, Rajendar V (2017). Nanocomposites of ZnO and TiO_2_ have enhanced antimicrobial and antibacterial properties than their disjoint counterparts. Dig J Nanomater Biostruct.

[139] El-Naggar ME, Shaheen TI, Zaghloul S, El-Rafie MH, Hebeish A (2016). Antibacterial activities and UV protection of the in situ synthesized titanium oxide nanoparticles on cotton fabrics. Ind Eng Chem Res.

[140] Tomak ED, Yazici OA, Parmak EDS, Gonultas O (2018). Influence of tannin containing coatings on weathering resistance of wood: combination with zinc and cerium oxide nanoparticles. Polym Degrad Stab.

[141] Li J, Wu Z, Bao Y, Chen Y, Huang C, Li N (2017). Wet chemical synthesis of ZnO nanocoating on the surface of bamboo timber with improved mould-resistance. J Saudi Chem Soc.

[142] Wang J, Li J, Zhuang X, Pan X, Yu H, Sun F (2018). Improved mould resistance and antibacterial activity of bamboo coated with ZnO/graphene. R Soc Open Sci.

[143] Weththimuni ML, Capsoni D, Malagodi M, Licchelli M (2019). Improving wood resistance to decay by nanostructured ZnO-based treatments. J Nanomater.

[144] Cheng D, Wen Y, An X, Zhu X, Ni Y (2016). TEMPO-oxidized cellulose nanofibers (TOCNs) as a green reinforcement for waterborne polyurethane coating (WPU) on wood. Carbohydr Polym.

[145] Cheng L, Ren S, Lu X (2020). Application of eco-friendly waterborne polyurethane composite coating incorporated with nano cellulose crystalline and silver nano particles on wood antibacterial board. Polymers (Basel).

[146] Boivin G, Ritcey AM, Landry V (2019). The effect of silver nanoparticles on the black-stain resistance of acrylic resin for translucent wood coating application. BioResources.

[147] Iždinský J, Reinprecht L, Nosál E (2018). Antibacterial efficiency of silver and zinc-oxide nanoparticles in acrylate coating for surface treatment of wooden composites. Wood Res.

[148] Ermeydan MA, Cabane E, Masic A, Koetz J, Burgert I (2012). Flavonoid insertion into cell walls improves wood properties. ACS Appl Mater Interfaces.

[149] Papadopoulos AN, Kyzas GZ (2019) Nanotechnology and wood science. In: Interface science and technology. Elsevier, London, pp 199–216

[150] Nikolic M, Lawther JM, Sanadi AR (2015). Use of nanofillers in wood coatings: a scientific review. J Coat Technol Res.

[151] Wu Y, Wu X, Yang F, Ye J (2020). Preparation and characterization of waterborne UV lacquer product modified by zinc oxide with flower shape. Polymers (Basel).

[152] Moya R, Rodríguez-Zúñiga A, Vega-Baudrit J, Puente-Urbina A (2017). Effects of adding TiO_2_ nanoparticles to a water-based varnish for wood applied to nine tropical woods of Costa Rica exposed to natural and accelerated weathering. J Coatings Technol Res.

[153] Huang J, Lyu S, Fu F, Wu Y, Wang S (2017). Green preparation of a cellulose nanocrystals/polyvinyl alcohol composite superhydrophobic coating. RSC Adv.

[154] Cataldi A, Corcione CE, Frigione M, Pegoretti A (2017). Photocurable resin/nanocellulose composite coatings for wood protection. Prog Org Coat.

[155] Xuan L, Fu Y, Liu Z, Wei P, Wu L (2018). Hydrophobicity and photocatalytic activity of a wood surface coated with a Fe^3+^-doped SiO_2_/TiO_2_ Film. Materials (Basel).

[156] Chang H, Tu K, Wang X, Liu J (2015). Fabrication of mechanically durable superhydrophobic wood surfaces using polydimethylsiloxane and silica nanoparticles. Rsc Adv.

[157] Gao L, Lu Y, Li J, Sun Q (2016). Superhydrophobic conductive wood with oil repellency obtained by coating with silver nanoparticles modified by fluoroalkyl silane. Holzforschung.

[158] Li J, Sun Q, Yao Q, Wang J, Han S, Jin C (2015). Fabrication of robust superhydrophobic bamboo based on ZnO nanosheet networks with improved water-, UV-, and fire-resistant properties. J Nanomater.

[159] Li J, Zheng H, Sun Q, Han S, Fan B, Yao Q (2015). Fabrication of superhydrophobic bamboo timber based on an anatase TiO_2_ film for acid rain protection and flame retardancy. RSC Adv.

[160] Zheng C, Wen S, Teng Z, Ye C, Chen Q, Zhuang Y (2019). Formation of H_2_Ti_2_O_5_·H_2_O nanotube-based hybrid coating on bamboo fibre materials through layer-by-layer self-assembly method for an improved flame retardant performance. Cellulose.

[161] Lu X, Hu Y (2016). Layer-by-layer deposition of TiO_2_ nanoparticles in the wood surface and its superhydrophobic performance. BioResources.

[162] Tu K, Wang X, Kong L, Guan H (2018). Facile preparation of mechanically durable, self-healing and multifunctional superhydrophobic surfaces on solid wood. Mater Des.

[163] Qing Y, Liu M, Wu Y, Jia S, Wang S, Li X (2017). Investigation on stability and moisture absorption of superhydrophobic wood under alternating humidity and temperature conditions. Results Phys.

[164] Havrlik M, Ryparová P (2015). Protection of wooden materials against biological attack by using nanotechnology. Acta Polytech.

[165] Papadopoulos AN, Taghiyari HR (2019). Innovative wood surface treatments based on nanotechnology. Coatings.

[166] Kong L, Xu D, He Z, Wang F, Gui S, Fan J (2019). Nanocellulose-reinforced polyurethane for waterborne wood coating. Molecules.

[167] Auclair N, Kaboorani A, Riedl B, Landry V, Hosseinaei O, Wang S (2018). Influence of modified cellulose nanocrystals (CNC) on performance of bionanocomposite coatings. Prog Org Coat.

[168] Meng L, Qiu H, Wang D, Feng B, Di M, Shi J (2020). Castor-oil-based waterborne acrylate/SiO_2_ hybrid coatings prepared via sol–gel and thiol-ene reactions. Prog Org Coat.

[169] Fallah F, Khorasani M, Ebrahimi M (2017). Improving the mechanical properties of waterborne nitrocellulose coating using nano-silica particles. Prog Org Coat.

[170] Guo S, Wang D, Shi J, Li X, Feng B, Meng L (2019). Study on waterborne acrylate coatings modified with biomass silicon. Prog Org Coat.

[171] Teacă CA, Roşu D, Bodîrlău R, Roşu L (2013). Structural changes in wood under artificial UV light irradiation determined by FTIR spectroscopy and color measurements—a brief review. BioResources.

[172] Andrady AL, Hamid H, Torikai A (2011). Effects of solar UV and climate change on materials. Photochem Photobiol Sci.

[173] Wallenhorst L, Gurău L, Gellerich A, Militz H, Ohms G, Viöl W (2018). UV-blocking properties of Zn/ZnO coatings on wood deposited by cold plasma spraying at atmospheric pressure. Appl Surf Sci.

[174] Rao F, Zhang Y, Bao M, Zhang Z, Bao Y, Li N (2019). Photostabilizing efficiency of acrylic-based bamboo exterior coatings combining benzotriazole and zinc oxide nanoparticles. Coatings.

[175] Pánek M, Oberhofnerová E, Hýsek Š, Šedivka P, Zeidler A (2018). Colour stabilization of oak, spruce, larch and douglas fir heartwood treated with mixtures of nanoparticle dispersions and UV-stabilizers after exposure to UV and VIS-radiation. Materials (Basel).

[176] Zheng R, Tshabalala MA, Li Q, Wang H (2015). Weathering performance of wood coated with a combination of alkoxysilanes and rutile TiO_2_ heirarchical nanostructures. BioResources.

[177] Zheng R, Tshabalala MA, Li Q, Wang H (2016). Photocatalytic degradation of wood coated with a combination of rutile TiO_2_ nanostructures and low-surface free-energy materials. BioResources.

[178] Deraman AF, Chandren S (2019) Fire-retardancy of wood coated by titania nanoparticles. In: AIP conference proceedings. AIP Publishing LLC, London, p 20022

[179] Ren D, Li J, Xu J, Wu Z, Bao Y, Li N (2018). Efficient antifungal and flame-retardant properties of ZnO–TiO_2_-layered double-nanostructures coated on bamboo substrate. Coatings.

[180] Yao X, Du C, Hua Y, Zhang J, Peng R, Huang Q (2019). Flame-retardant and smoke suppression properties of nano MgAl-LDH coating on bamboo prepared by an in situ reaction. J Nanomater.

[181] Wang X, Kalali EN, Xing W, Wang D-Y (2018). CO_2_ induced synthesis of Zn–Al layered double hydroxide nanostructures towards efficiently reducing fire hazards of polymeric materials. Nano Adv.

[182] Esmailpour A, Majidi R, Taghiyari HR, Ganjkhani M, Mohseni Armaki SM, Papadopoulos AN (2020). Improving fire retardancy of beech wood by graphene. Polymers (Basel).

[183] Hunt D (2012). Properties of wood in the conservation of historical wooden artifacts. J Cult Herit.

[184] Mazzanti P, Togni M, Uzielli L (2012). Drying shrinkage and mechanical properties of poplar wood (Populus alba L.) across the grain. J Cult Herit.

[185] Švajlenka J, Kozlovská M (2020). Evaluation of the efficiency and sustainability of timber-based construction. J Clean Prod.

[186] Ajuong E, Pinion LC, Bhuiyan MSH (2018) Degradation of wood. In: Reference module in materials science and materials engineering. Elsevier Inc. 10.1016/B978-0-12-803581-8.10537-5

[187] Bari E, Daryaei MG, Karim M, Bahmani M, Schmidt O, Woodward S (2019). Decay of Carpinus betulus wood by Trametes versicolor—an anatomical and chemical study. Int Biodeterior Biodegrad.

[188] Zabel RA, Morrell JJ (1992). Wood microbiology: decay and its prevention.

[189] Khan AAH, Karuppayil SM (2012). Fungal pollution of indoor environments and its management. Saudi J Biol Sci.

[190] Oliveira GL, de Oliveira FL, Brazolin S (2018). Wood preservation for preventing biodeterioration of cross laminated timber (CLT) panels assembled in tropical locations. Proc Struct Integr.

[191] Kumar S, Bhanjana G, Sharma A, Sidhu MC, Dilbaghi N (2014). Synthesis, characterization and on field evaluation of pesticide loaded sodium alginate nanoparticles. Carbohydr Polym.

[192] Sanders F (2008) Reregistration eligibility decision for chromated arsenicals (List A Case No. 0132). EPA 739-R-08-00. United States Environmental Protection Agency, Washington, DC

[193] Mercer TG, Frostick LE (2014). Evaluating the potential for environmental pollution from chromated copper arsenate (CCA)-treated wood waste: a new mass balance approach. J Hazard Mater.

[194] Ohgami N, Yamanoshita O, Thang ND, Yajima I, Nakano C, Wenting W (2015). Carcinogenic risk of chromium, copper and arsenic in CCA-treated wood. Environ Pollut.

[195] Krause C, Chutsch M, Englert N (1989). Pentachlorophenol exposure through indoor use of wood preservatives in the Federal Republic of Germany. Environ Int.

[196] Yang L, Nyalwidhe JO, Guo S, Drake RR, Semmes OJ (2011). Targeted identification of metastasis-associated cell-surface sialoglycoproteins in prostate cancer. Mol Cell Proteomics (Internet).

[197] Mantanis G, Terzi E, Kartal SN, Papadopoulos AN (2014). Evaluation of mold, decay and termite resistance of pine wood treated with zinc-and copper-based nanocompounds. Int Biodeterior Biodegrad.

[198] Papadopoulos AN, Bikiaris DN, Mitropoulos AC, Kyzas GZ (2019). Nanomaterials and chemical modifications for enhanced key wood properties: a review. Nanomaterials.

[199] Goffredo GB, Accoroni S, Totti C, Romagnoli T, Valentini L, Munafò P (2017). Titanium dioxide based nanotreatments to inhibit microalgal fouling on building stone surfaces. Build Environ.

[200] Hill CAS, Papadopoulos AN (2001). A review of methods used to determine the size of the cell wall microvoids of wood. J Inst Wood Sci..

[201] Teng T-J, Arip MNM, Sudesh K, Nemoikina A, Jalaludin Z, Ng E-P (2018). Conventional technology and nanotechnology in wood preservation: a review. BioResources.

[202] Margulis-Goshen K, Magdassi S (2013) Nanotechnology: an advanced approach to the development of potent insecticides. In: Advanced technologies for managing insect pests. Springer, Berlin, pp 295–314

[203] Nair R, Varghese SH, Nair BG, Maekawa T, Yoshida Y, Kumar DS (2010). Nanoparticulate material delivery to plants. Plant Sci.

[204] Houston S, Hof R, Francescutti T, Hawkes A, Boulanger MJ, Cameron CE (2011). Bifunctional role of the Treponema pallidum extracellular matrix binding adhesin Tp0751. Infect Immun (Internet).

[205] Gu Z, Wang M, Fang Q, Zheng H, Wu F, Lin D (2015). Preparation and in vitro characterization of pluronic-attached polyamidoamine dendrimers for drug delivery. Drug Dev Ind Pharm.

[206] Khoee S, Hashemi A, Molavipordanjani S (2018). Synthesis and characterization of IUdR loaded PEG/PCL/PEG polymersome in mixed DCM/DMF solvent: experimental and molecular dynamics insights into the role of solvent composition and star architecture in drug dispersion and diffusion. Eur J Pharm Sci.

[207] Lee M-Y, Min S-G, You S-K, Choi M-J, Hong G-P, Chun J-Y (2013). Effect of β-cyclodextrin on physical properties of nanocapsules manufactured by emulsion–diffusion method. J Food Eng.

[208] Nabi-Meibodi M, Navidi B, Navidi N, Vatanara A, Rouini MR, Ramezani V (2013). Optimized double emulsion-solvent evaporation process for production of solid lipid nanoparticles containing baclofene as a lipid insoluble drug. J Drug Deliv Sci Technol.

[209] Mattos BD, Tardy BL, Magalhães WLE, Rojas OJ (2017). Controlled release for crop and wood protection: Recent progress toward sustainable and safe nanostructured biocidal systems. J Control Release.

[210] Kumar S, Chauhan N, Gopal M, Kumar R, Dilbaghi N (2015). Development and evaluation of alginate–chitosan nanocapsules for controlled release of acetamiprid. Int J Biol Macromol.

[211] Ding X, Richter DL, Matuana LM, Heiden PA (2011). Efficient one-pot synthesis and loading of self-assembled amphiphilic chitosan nanoparticles for low-leaching wood preservation. Carbohydr Polym.

[212] Bai C, Zhang S, Huang L, Wang H, Wang W, Ye Q (2015). Starch-based hydrogel loading with carbendazim for controlled-release and water absorption. Carbohydr Polym.

[213] Mattos BD, Magalhães WLE (2016). Biogenic nanosilica blended by nanofibrillated cellulose as support for slow-release of tebuconazole. J Nanoparticle Res.

[214] Jämsä S, Mahlberg R, Holopainen U, Ropponen J, Savolainen A, Ritschkoff A-C (2013). Slow release of a biocidal agent from polymeric microcapsules for preventing biodeterioration. Prog Org Coat.

[215] Qian K, Shi T, He S, Luo L, Cao Y (2013). Release kinetics of tebuconazole from porous hollow silica nanospheres prepared by miniemulsion method. Microporous Mesoporous Mater.

[216] Collin JP, Durot S, Keller M, Sauvage JP, Trolez Y, Cetina M (2011). Synthesis of [5]rotaxanes containing Bi- and tridentate coordination sites in the axis. Chem A Eur J (Internet).

[217] Can A, Sivrikaya H, Hazer B (2018). Fungal inhibition and chemical characterization of wood treated with novel polystyrene-soybean oil copolymer containing silver nanoparticles. Int Biodeterior Biodegrad.

[218] Künniger T, Gerecke AC, Ulrich A, Huch A, Vonbank R, Heeb M (2014). Release and environmental impact of silver nanoparticles and conventional organic biocides from coated wooden façades. Environ Pollut.

[219] Lykidis C, Bak M, Mantanis G, Németh R (2016). Biological resistance of pine wood treated with nano-sized zinc oxide and zinc borate against brown-rot fungi. Eur J Wood Wood Prod.

[220] Mantanis GI, Papadopoulos AN (2010). The sorption of water vapour of wood treated with a nanotechnology compound. Wood Sci Technol.

[221] Green F, Arango RA (2007) Wood protection by commercial silver formulations against Eastern subterranean termites. IRG/WP; 07-30422 Stock Sweden IRG Secr 2007, p 6

[222] Freeman MH, Mcintyre CR (2013). Micronized copper wood preservatives: Strong indications of the reservoir effect. Int Res Gr Wood Prot..

[223] Xue W, Kennepohl P, Ruddick W (2014) Chemistry of copper preservative treated wood. In: Proc 45th Int Gr Wood Prot St Georg UT, USA, pp 11–15

[224] Freeman MH, McIntyre CR (2008). Copper-based wood preservatives. For Prod J.

[225] Oliva R, Salvini A, Di Giulio G, Capozzoli L, Fioravanti M, Giordano C (2015). TiO_2_-Oligoaldaramide nanocomposites as efficient core–shell systems for wood preservation. J Appl Polym Sci.

[226] Vučetić SB, Rudić OL, Markov SL, Bera OJ, Vidaković AM, Skapin ASS (2014). Antifungal efficiency assessment of the TiO_2_ coating on façade paints. Environ Sci Pollut Res.

[227] Afrouzi YM, Marzbani P, Omidvar A (2015). The effect of moisture content on the retention and distribution of nano-titanium dioxide in the wood. Maderas Cienc Tecnol.

[228] Harandi D, Ahmadi H, Achachluei MM (2016). Comparison of TiO_2_ and ZnO nanoparticles for the improvement of consolidated wood with polyvinyl butyral against white rot. Int Biodeterior Biodegrad.

[229] Nair S, Nagarajappa GB, Pandey KK (2018). UV stabilization of wood by nano metal oxides dispersed in propylene glycol. J Photochem Photobiol B Biol.

[230] Kartal SN, Green Iii F, Clausen CA (2009). Do the unique properties of nanometals affect leachability or efficacy against fungi and termites?. Int Biodeterior Biodegrad.

[231] Mohammadnia-Afrouzi Y, Marzbani P, Ahmadinejad A (2014). Investigation on the weathering resistance of poplar wood impregnated with nano ZnO–AgO Mixture. Adv Environ Biol.

[232] Terzi E, Coşkun K, Kartal SN (2019). Mold resistance of nano and micronized particles-treated wood after artificial weathering process. J Anatol Environ Anim Sci.

[233] Echiegu EA (2016). Nanotechnology as a tool for enhanced renewable energy application in developing countries. J Fundam Renew Energy Appl.

[234] Serrano E, Rus G, Garcia-Martinez J (2009). Nanotechnology for sustainable energy. Renew Sustain Energy Rev.

[235] Hut I, Pelemiš SS, Mirjanić DL (2015). Nanomaterials and nanotechnology for sustainable energy. Zaštita Mater.

[236] Raghav SB, Dinesh V (2016). Recent developments on nanotechnology in solar energy. Int J Eng Comput Sci.

[237] Du X, Zhang Z, Liu W, Deng Y (2017). Nanocellulose-based conductive materials and their emerging applications in energy devices—a review. Nano Energy.

[238] Deshmukh P, Katariya S (2013). Nanotechnology applications in the energy sector. Int J Adv Res Technol.

[239] Rani K, Sridevi V (2017). An overview on role of nanotechnology in green and clean technology. Austin Env Sci.

[240] Delgado J, Öchsner A, de Lima AG (2014) Nanotechnology for energy and environment. Hindawi

[241] Jung M, Kim K, Kim B, Lee K-J, Kang J-W, Jeon S (2017). Vertically stacked nanocellulose tactile sensor. Nanoscale.

[242] Vicente AT, Araújo A, Mendes MJ, Nunes D, Oliveira MJ, Sanchez-Sobrado O (2018). Multifunctional cellulose-paper for light harvesting and smart sensing applications. J Mater Chem C.

[243] Klochko NP, Barbash VA, Klepikova KS, Kopach VR, Tyukhov II, Yashchenko OV (2020). Use of biomass for a development of nanocellulose-based biodegradable flexible thin film thermoelectric material. Sol Energy.

[244] Hsu HH, Zhong W (2019). Nanocellulose-based conductive membranes for free-standing supercapacitors: a review. Membranes (Basel).

[245] Koga H, Saito T, Kitaoka T, Nogi M, Suganuma K, Isogai A (2013). Transparent, conductive, and printable composites consisting of TEMPO-oxidized nanocellulose and carbon nanotube. Biomacromol.

[246] Tian X, Yang C, Si L, Si G (2017). Flexible self-assembled membrane electrodes based on eco-friendly bamboo fibers for supercapacitors. J Mater Sci Mater Electron.

[247] Wang Z, Tammela P, Zhang P, Strømme M, Nyholm L (2014). Efficient high active mass paper-based energy-storage devices containing free-standing additive-less polypyrrole–nanocellulose electrodes. J Mater Chem A.

[248] Wang Z, Tammela P, Zhang P, Huo J, Ericson F, Strømme M (2014). Freestanding nanocellulose-composite fibre reinforced 3D polypyrrole electrodes for energy storage applications. Nanoscale.

[249] Tammela P, Wang Z, Frykstrand S, Zhang P, Sintorn I-M, Nyholm L (2015). Asymmetric supercapacitors based on carbon nanofibre and polypyrrole/nanocellulose composite electrodes. Rsc Adv.

[250] Razaq A, Nyholm L, Sjödin M, Strømme M, Mihranyan A (2012). Paper-based energy-storage devices comprising carbon fiber-reinforced polypyrrole-cladophora nanocellulose composite electrodes. Adv Energy Mater.

[251] Dunn B, Kamath H, Tarascon J-M (2011). Electrical energy storage for the grid: a battery of choices. Science (80–).

[252] Lin L, Ning H, Song S, Xu C, Hu N (2020). Flexible electrochemical energy storage: the role of composite materials. Compos Sci Technol.

[253] Quartarone E, Mustarelli P (2011). Electrolytes for solid-state lithium rechargeable batteries: recent advances and perspectives. Chem Soc Rev.

[254] Xu K (2014). Electrolytes and interphases in Li-ion batteries and beyond. Chem Rev.

[255] Janek J, Zeier WG (2016). A solid future for battery development. Nat Energy.

[256] Qin H, Fu K, Zhang Y, Ye Y, Song M, Kuang Y (2020). Flexible nanocellulose enhanced Li^+^ conducting membrane for solid polymer electrolyte. Energy Storage Mater.

[257] Nair JR, Bella F, Angulakshmi N, Stephan AM, Gerbaldi C (2016). Nanocellulose-laden composite polymer electrolytes for high performing lithium–sulphur batteries. Energy Storage Mater.

[258] Zhang Y, Zhong Y, Bian W, Liao W, Zhou X, Jiang F (2020). Robust proton exchange membrane for vanadium redox flow batteries reinforced by silica-encapsulated nanocellulose. Int J Hydrog Energy.

[259] Parasuraman A, Lim TM, Menictas C, Skyllas-Kazacos M (2013). Review of material research and development for vanadium redox flow battery applications. Electrochim Acta.

[260] Pérez-Madrigal MM, Edo MG, Alemán C (2016). Powering the future: application of cellulose-based materials for supercapacitors. Green Chem.

[261] Him NRN, Apau C, Azmi NS (2016). Effect of temperature and pH on deinking of laser-jet waste paper using commercial lipase and esterase. J Liife Sci Technol.

[262] Gui Z, Zhu H, Gillette E, Han X, Rubloff GW, Hu L (2013). Natural cellulose fiber as substrate for supercapacitor. ACS Nano.

[263] Nie S, Zhang K, Lin X, Zhang C, Yan D, Liang H (2018). Enzymatic pretreatment for the improvement of dispersion and film properties of cellulose nanofibrils. Carbohydr Polym.

[264] Yao K, Meng Q, Bulone V, Zhou Q (2017). Flexible and responsive chiral nematic cellulose nanocrystal/poly (ethylene glycol) composite films with uniform and tunable structural color. Adv Mater.

[265] Khosrozadeh A, Ali Darabi M, Xing M, Wang Q (2015). Flexible cellulose-based films of polyaniline–Graphene–Silver nanowire for high-performance supercapacitors. J Nanotechnol Eng Med.

[266] Ma L, Liu R, Niu H, Wang F, Liu L, Huang Y (2016). Freestanding conductive film based on polypyrrole/bacterial cellulose/graphene paper for flexible supercapacitor: large areal mass exhibits excellent areal capacitance. Electrochim Acta.

[267] Gao K, Shao Z, Wu X, Wang X, Li J, Zhang Y (2013). Cellulose nanofibers/reduced graphene oxide flexible transparent conductive paper. Carbohydr Polym.

[268] An Y, Yang Y, Hu Z, Guo B, Wang X, Yang X (2017). High-performance symmetric supercapacitors based on carbon nanosheets framework with graphene hydrogel architecture derived from cellulose acetate. J Power Sources.

[269] Zheng G, Cui Y, Karabulut E, Wågberg L, Zhu H, Hu L (2013). Nanostructured paper for flexible energy and electronic devices. MRS Bull.

[270] Li S, Lee PS (2017). Development and applications of transparent conductive nanocellulose paper. Sci Technol Adv Mater.

[271] Sabo R, Yermakov A, Law CT, Elhajjar R (2016). Nanocellulose-enabled electronics, energy harvesting devices, smart materials and sensors: a review. J Renew Mater.

[272] Okahisa Y, Yoshida A, Miyaguchi S, Yano H (2009). Optically transparent wood–cellulose nanocomposite as a base substrate for flexible organic light-emitting diode displays. Compos Sci Technol.

[273] Jiang Y, Sun D-W, Pu H, Wei Q (2018). Surface enhanced Raman spectroscopy (SERS): a novel reliable technique for rapid detection of common harmful chemical residues. Trends Food Sci Technol.

[274] Li Y, Wang Z, Sun L, Liu L, Xu C, Kuang H (2019). Nanoparticle-based sensors for food contaminants. TrAC Trends Anal Chem.

[275] Dungchai W, Chailapakul O, Henry CS (2009). Electrochemical detection for paper-based microfluidics. Anal Chem.

[276] Suaifan GARY, Esseghaier C, Ng A, Zourob M (2013). Ultra-rapid colorimetric assay for protease detection using magnetic nanoparticle-based biosensors. Analyst.

[277] Sharma R, Ragavan KV, Thakur MS, Raghavarao K (2015). Recent advances in nanoparticle based aptasensors for food contaminants. Biosens Bioelectron.

[278] Portaccio M, Durante D, Viggiano A, Di Martino S, De Luca P, Di Tuoro D (2007). Amperometric glucose determination by means of glucose oxidase immobilized on a cellulose acetate film: dependence on the immobilization procedures. Electroanal An Int J Devoted to Fundam Pract Asp Electroanal.

[279] Wang M, Anoshkin IV, Nasibulin AG, Korhonen JT, Seitsonen J, Pere J (2013). Modifying native nanocellulose aerogels with carbon nanotubes for mechanoresponsive conductivity and pressure sensing. Adv Mater.

[280] Yan C, Wang J, Kang W, Cui M, Wang X, Foo CY (2014). Highly stretchable piezoresistive graphene–nanocellulose nanopaper for strain sensors. Adv Mater.

[281] Naghdi T, Golmohammadi H, Vosough M, Atashi M, Saeedi I, Maghsoudi MT (2019). Lab-on-nanopaper: an optical sensing bioplatform based on curcumin embedded in bacterial nanocellulose as an albumin assay kit. Anal Chim Acta.

[282] Ling Z, Xu F, Edwards JV, Prevost NT, Nam S, Condon BD (2019). Nanocellulose as a colorimetric biosensor for effective and facile detection of human neutrophil elastase. Carbohydr Polym.

[283] Abdi MM, Razalli RL, Tahir PM, Chaibakhsh N, Hassani M, Mir M (2019). Optimized fabrication of newly cholesterol biosensor based on nanocellulose. Int J Biol Macromol (Internet).

[284] Yao Y, Huang X, Zhang B, Zhang Z, Hou D, Zhou Z (2020). Facile fabrication of high sensitivity cellulose nanocrystals based QCM humidity sensors with asymmetric electrode structure. Sensors Actuators B Chem.

[285] Ng HKM, Lim GK, Leo CP (2020). N-modified carbon quantum dot in 3D-network of microfibrillated cellulose for building photoluminescent thin film as tartrazine sensor. J Photochem Photobiol A Chem.

[286] Kim HJ, Park S, Kim SH, Kim JH, Yu H, Kim HJ (2015). Biocompatible cellulose nanocrystals as supports to immobilize lipase. J Mol Catal B Enzym.

[287] Edwards JV, Prevost NT, French AD, Concha M, Condon BD (2015). Kinetic and structural analysis of fluorescent peptides on cotton cellulose nanocrystals as elastase sensors. Carbohydr Polym.

[288] Incani V, Danumah C, Boluk Y (2013). Nanocomposites of nanocrystalline cellulose for enzyme immobilization. Cellulose.

[289] Abd Manan FA, Hong WW, Abdullah J, Yusof NA, Ahmad I (2019). Nanocrystalline cellulose decorated quantum dots based tyrosinase biosensor for phenol determination. Mater Sci Eng C.

[290] Moradi M, Tajik H, Almasi H, Forough M, Ezati P (2019). A novel pH-sensing indicator based on bacterial cellulose nanofibers and black carrot anthocyanins for monitoring fish freshness. Carbohydr Polym.

[291] Ruiz-Palomero C, Benítez-Martínez S, Soriano ML, Valcárcel M (2017). Fluorescent nanocellulosic hydrogels based on graphene quantum dots for sensing laccase. Anal Chim Acta.

[292] Jeon J, Kim E, Murugesan K, Park H, Kim Y, Kwon J (2010). Laccase-catalysed polymeric dye synthesis from plant-derived phenols for potential application in hair dyeing: enzymatic colourations driven by homo-or hetero-polymer synthesis. Microb Biotechnol.

[293] Li Y, Greenwall L (2013). Safety issues of tooth whitening using peroxide-based materials. Br Dent J.

[294] Faham S, Golmohammadi H, Ghavami R, Khayatian G (2019). A nanocellulose-based colorimetric assay kit for smartphone sensing of iron and iron-chelating deferoxamine drug in biofluids. Anal Chim Acta.

[295] Burrs SL, Bhargava M, Sidhu R, Kiernan-Lewis J, Gomes C, Claussen JC (2016). A paper based graphene-nanocauliflower hybrid composite for point of care biosensing. Biosens Bioelectron.

[296] Burrs SL, Vanegas DC, Bhargava M, Mechulan N, Hendershot P, Yamaguchi H (2015). A comparative study of graphene–hydrogel hybrid bionanocomposites for biosensing. Analyst.

[297] Ortolani TS, Pereira TS, Assumpção MHMT, Vicentini FC, de Oliveira GG, Janegitz BC (2019). Electrochemical sensing of purines guanine and adenine using single-walled carbon nanohorns and nanocellulose. Electrochim Acta.

[298] Shalauddin M, Akhter S, Basirun WJ, Bagheri S, Anuar NS, Johan MR (2019). Hybrid nanocellulose/f-MWCNTs nanocomposite for the electrochemical sensing of diclofenac sodium in pharmaceutical drugs and biological fluids. Electrochim Acta.

[299] Blaise C, Gagné F, Ferard JF, Eullaffroy P (2008). Ecotoxicity of selected nano-materials to aquatic organisms. Environ Toxicol An Int J.

[300] Ong KJ, Ede JD, Pomeroy-Carter CA, Sayes CM, Mulenos MR, Shatkin JA (2020). A 90-day dietary study with fibrillated cellulose in Sprague–Dawley rats. Toxicol Rep.

[301] McClements DJ, Xiao H (2017). Is nano safe in foods? Establishing the factors impacting the gastrointestinal fate and toxicity of organic and inorganic food-grade nanoparticles. NPJ Sci Food.

[302] Poulsen SS, Jackson P, Kling K, Knudsen KB, Skaug V, Kyjovska ZO (2016). Multi-walled carbon nanotube physicochemical properties predict pulmonary inflammation and genotoxicity. Nanotoxicology.

[303] O’Connor B, Berry R, Goguen R (2014) Commercialization of cellulose nanocrystal (NCC^TM^) production: a business case focusing on the importance of proactive EHS management. In: Nanotechnology environmental health and safety. Elsevier, London, pp 225–246

[304] Edgar KJ, Heinze T, Buchanan CM (2009). Polysaccharide materials: performance by design.

[305] Villanova JCO, Ayres E, Carvalho SM, Patrício PS, Pereira FV, Oréfice RL (2011). Pharmaceutical acrylic beads obtained by suspension polymerization containing cellulose nanowhiskers as excipient for drug delivery. Eur J Pharm Sci.

[306] Male KB, Leung ACW, Montes J, Kamen A, Luong JHT (2012). Probing inhibitory effects of nanocrystalline cellulose: inhibition versus surface charge. Nanoscale.

[307] Dong S, Hirani AA, Colacino KR, Lee YW, Roman M (2012). Cytotoxicity and cellular uptake of cellulose nanocrystals. Nano Life.

[308] Yang X, Bakaic E, Hoare T, Cranston ED (2013). Injectable polysaccharide hydrogels reinforced with cellulose nanocrystals: morphology, rheology, degradation, and cytotoxicity. Biomacromol.

[309] Hanif Z, Ahmed FR, Shin SW, Kim Y-K, Um SH (2014). Size-and dose-dependent toxicity of cellulose nanocrystals (CNC) on human fibroblasts and colon adenocarcinoma. Colloids Surfaces B Biointerfaces.

[310] Åkerlund E, Islam MS, McCarrick S, Alfaro-Moreno E, Karlsson HL (2019). Inflammation and (secondary) genotoxicity of Ni and NiO nanoparticles. Nanotoxicology.

[311] Lapied E, Nahmani JY, Moudilou E, Chaurand P, Labille J, Rose J (2011). Ecotoxicological effects of an aged TiO_2_ nanocomposite measured as apoptosis in the anecic earthworm Lumbricus terrestris after exposure through water, food and soil. Environ Int.

[312] Van HK, Quik JTK, Mankiewicz-Boczek J, De SKAC, Elsaesser A, Van der Meeren P (2009). Fate and effects of CeO_2_ nanoparticles in aquatic ecotoxicity tests. Environ Sci Technol.

[313] Pulido-Reyes G, Rodea-Palomares I, Das S, Sakthivel TS, Leganes F, Rosal R (2015). Untangling the biological effects of cerium oxide nanoparticles: the role of surface valence states. Sci Rep.

[314] Wang Z, Song L, Ye N, Yu Q, Zhai Y, Zhang F (2020). Oxidative stress actuated by cellulose nanocrystals and nanofibrils in aquatic organisms of different trophic levels. NanoImpact.

[315] Vatta LL, Sanderson RD, Koch KR (2006). Magnetic nanoparticles: properties and potential applications. Pure Appl Chem.

[316] Mouchet F, Landois P, Flahaut E, Pinelli E, Gauthier L (2007). Assessment of the potential in vivo ecotoxicity of double-walled carbon nanotubes (DWNTs) in water, using the amphibian Ambystoma mexicanum. Nanotoxicology.

